# Host cytosolic RNA sensing pathway promotes T Lymphocyte-mediated mycobacterial killing in macrophages

**DOI:** 10.1371/journal.ppat.1008569

**Published:** 2020-05-28

**Authors:** Yong Cheng, Nicholas J. Kiene, Alexandra Tatarian, Emily F. Eix, Jeffrey S. Schorey

**Affiliations:** Department of Biological Sciences, Eck Institute for Global Health, Center for Rare and Neglected Diseases, University of Notre Dame, Notre Dame, Indiana, United States of America; University of Washington, UNITED STATES

## Abstract

Mycobacterial infection leads to activation of the RIG-I/MAVS/TBK1 RNA sensing pathway in macrophages but the consequences of this activation remains poorly defined. In this study, we determined that activation of this RNA sensing pathway stimulates ICAM-1 expression in *M*.*avium*-infected macrophage through the inhibition of the E3 ubiquitin ligase CRL4^COP1/DET1^. CRL4 when active targets the transcription factor ETV5 for degradation by the ubiquitin–proteasome system. In the absence of the ETV5 transcription factor, ICAM-1 expression is significantly decreased. The *M*.*avium*-induced ICAM-1 production is required for the formation of immune synapse between infected macrophages and antigen-specific CD4^+^ T lymphocytes, and is essential for CD4^+^ T lymphocyte-mediated mycobacterial killing in vitro and in mice. This study demonstrates a previously undefined mechanism by which a host cytosolic RNA sensing pathway contributes to the interplay between mycobacteria infected macrophages and antigen-specific T lymphocytes.

## Author summary

As intracellular pathogens mycobacteria illicit a T cell response that is critical for controlling the infection. T cell activation requires not only MHC/peptide and T cell receptor binding but various other receptor-ligand interactions. In the present study we found that activation of the RNA sensing pathway in macrophages infected with *M*. *avium* leads to the activation of the transcription factor ETV5 resulting in ICAM-1 expression. ICAM-1 is a known ligand for the T cell LFA-1. We found that the mycobacterial RNA induced expression of ICAM-1 was required for CD4+ T cell binding to *M*. *avium*-infected macrophages and for the T cell-mediated killing of the intracellular mycobacteria. Moreover, we found mice deficient in MAVS, a protein required for activation of the cytosolic RNA sensing pathway, were limited in their ability to control an *M*. *avium* infection. This loss of control was linked to the loss of ICAM-1 expression by infected alveolar macrophages. In summary, we demonstrate a previously undefined mechanism by which a host cytosolic RNA sensing pathway contributes to the interplay between mycobacteria infected macrophages and antigen-specific T lymphocytes.

## Introduction

Non-tuberculous mycobacteria (NTM) are opportunistic pathogens, predominantly causing pulmonary infections in susceptible populations including the elderly and in patients receiving immunosuppressive drugs, or with pre-existing conditions such as cystic fibrosis, chronic obstructive pulmonary disease (COPD) or bronchiectasis. The most commonly isolated NTM species are *Mycobacterium avium* complex (MAC) (*M*.*avium* and *Mycobacterium intracellulare*) and *Mycobacterium abscessus* complex (MABSC) (*M*. *abscessus*, *M*. *massiliense* and *M*. *bolletii*), which account for >90% of the total cases reported [1]. NTM infections are increasing in the U.S., Japan and many European countries. Much of this growth is due to increased infections of older Caucasian and Asian women who have no known underlying lung disease [2]. Despite their increased clinical importance we still have a limited knowledge of *M*.*avium* pathogenesis and host immunity.

In this study, we investigated the function of the cytosolic RNA sensing pathway during *M*. *avium* infection *in vitro* and *in vivo*. Host cytosolic RNA sensing pathways play a central role in detecting foreigner DNA and RNA in virally infected cells [3]. Recently, it was found that *Listeria monocytogenes* and *Mycobacterium tuberculosis* (*M*.*tb*) release their RNAs into host cells through the bacterial SecA2 secretion system and induce RIG-I/MAVS/TBK1-dependent type I IFN production [4,5]. However, whether the activation of the RIG-I/MAVS RNA sensor pathway stimulates other cellular responses following a bacterial infection remains undefined. During viral infections induction of the RIG-I/MAVS pathway has been shown to drive production of IL-1β and IL-18 [6], and promote release of cytochrome c from the mitochondria in a Bax-dependent manner leading to apoptosis of the infected cell [7]. These and other studies have demonstrated the importance of the RNA sensing pathway in promoting a protective host response to viral infections. However, the importance of RNA sensing pathways in the context of a bacterial infection is less clear and evidence points to both a protective and detrimental effect [5,8]. In support of the former we have identified a RIG-I/MAVS-dependent production of the Intercellular Adhesion Molecule 1 (ICAM-1) in macrophages following an *M*.*avium* infection.

ICAM-1 promotes cell-cell interaction by serving as the ligand for the leukocyte adhesion protein LFA-1 and Mac-1 [9]. It is also important in formation of immune synapse between T cells and antigen presenting cells (APCs) [10]. Expression of ICAM-1 is required to control an *M*. *avium* infection [11]. However, these studies were carried out using *ICAM-1*^*-/-*^ mice so ICAM-1’s role in T cell-APC interaction during a mycobacterial infection has not been defined. Moreover, what regulates ICAM-1 expression during a mycobacterial infection remains unclear. Previous studies have shown activation of signaling pathways such as PI3K/Akt and the MAPKs result in activation of the transcription factors AP-1 and NF-ƙB driving ICAM-1 expression [12]. Initiation of these pathways can be induced by engagement of receptors such as TNFR1, TLR4 and EGFR, among others [13]. In the present study we identified a previously unknown role for the transcription factor ETV5 in regulating ICAM-1 expression. We also found that ICAM-1 expression was essential for CD4^+^ T-mediated *M*.*avium* killing within infected macrophages and mice. Our study sheds new light on the host cytosolic RNA sensing pathway in controlling an NTM infection and identified a previously undefined role for the RIG-I/MAVS/TBK1 RNA sensing pathway in regulating the immune synapse between macrophage and CD4^+^ T cells, and subsequent macrophage activation by CD4^+^ T cells.

## Results

### *M*.*avium* activates the host cytosolic RIG-I/MAVS/TBK1/IRF3/IRF7 RNA sensing pathway

**Fig 1:**
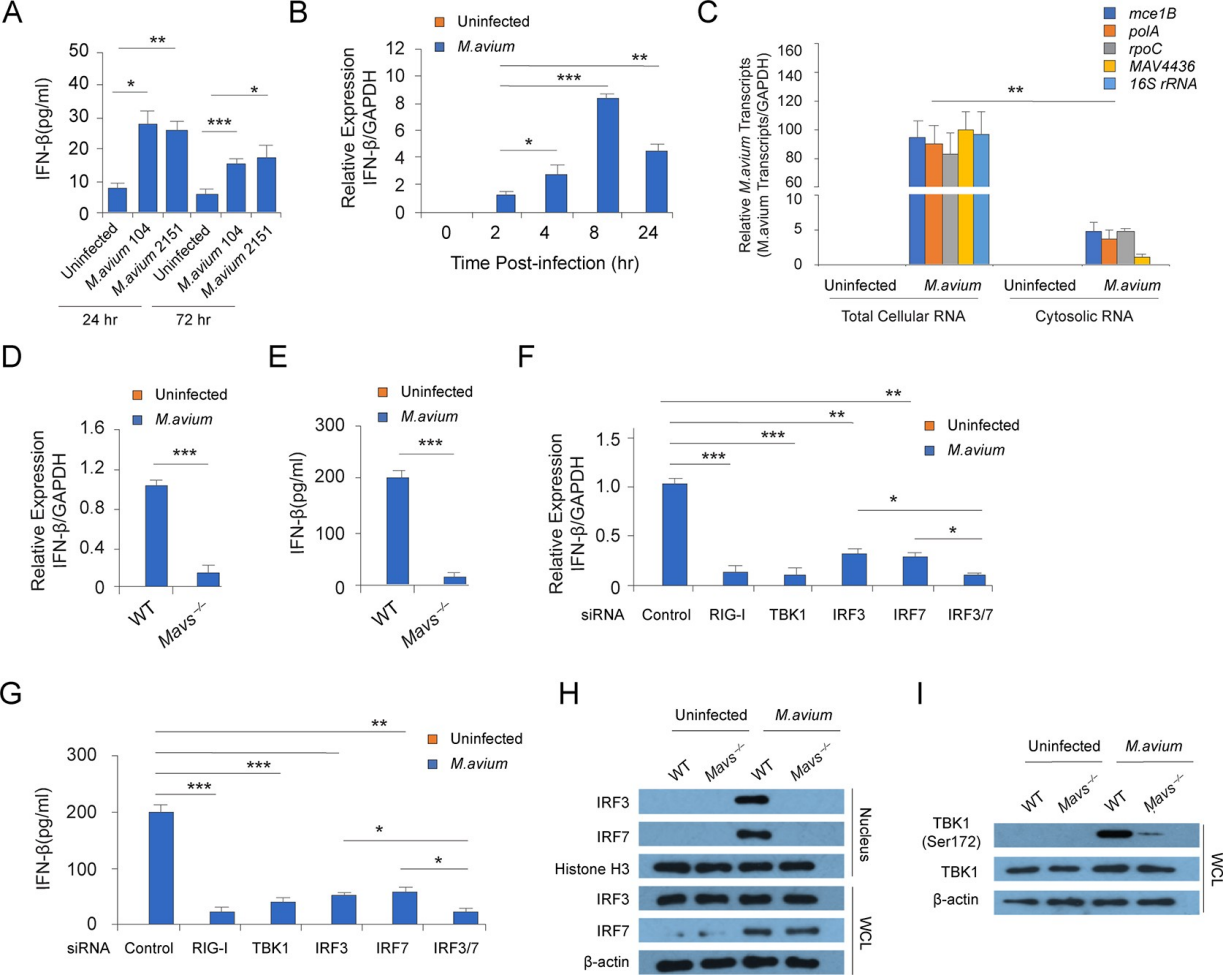
*M*. *avium* induces IFN-β production in macrophages through a cytosolic RIG-I/MAVS/TBK1/IRF3/7-depdendent pathway. (A) ELISA analysis for IFN-β production in WT BMMs infected with *M*. *avium* 104 and 2151 at 24 and 72 hr post-infection. (B) qRT-PCR analysis for IFN-β production in WT BMMs infected with *M*. *avium* 104 at various time points post-infection. The IFN-β mRNA levels were expressed as fold change relative to *M*. *avium*-infected BMMs at 2 hr post-infection. (C) qRT-PCR analysis for mycobacterial RNA abundancy in *M*. *avium*-infected BMMs (Total Cellular RNA) or the cytosol (Cytosolic RNA) at 24 hr post-infection. Each *M*. *avium* 104 transcript abundance was expressed as the percentage relative to *M*. *avium*-infected BMMs (Total Cellular RNA). (D) qRT-PCR analysis for IFN-β production in *M*. *avium*-infected WT and *Mavs*^*-/-*^ BMMs at 24 hr post-infection. (E) Similar to (D), but IFN-β protein was quantified by ELISA. (F) Similar to (D), but *M*.*avium*-induced IFN-β production was determined in BMMs at 24 hr post-infection following a 48 hr pretreated with negative control (Control), RIG-I, TBK1, IRF3 or IRF7 siRNA. (G) Similar to (F), but *M*.*avium*-induced IFN-β production was measured by ELISA. (H) Western Blot analysis for IRF3 and IRF7 in whole cell lysate (WCL) and nuclear fraction (Nucleus) of WT and *Mavs*^*-/-*^ BMMs at 24 hr post-infection. (I) Similar to (H), but TBK1 phosphorylation (Ser172) was analyzed in WCL. β-Actin and Histone H3 served as loading control for WCL and nuclear fraction, respectively. Data shown in (A-G) are the mean ±SD (n = 3 wells per group), and all data are representative of three independent experiments (biological replicates). For (D) and (F), the data was expressed as fold change relative to *M*.*avium*-infected WT (D) or control (F) BMMs. GAPDH served as loading control in qRT-PCR. *P < 0.05, **P < 0.01, and ***P < 0.001 by Student’s t-test (two-tailed).

Our previous study showed that *M*.*tb* release mycobacterial RNA into the cytosol of infected macrophages via a mycobacterial SecA2-dependent pathway. Mycobacterial species including *M*.*avium* also express a SecA2 secretion system. The *M*.*avium* SecA2 shares 93% homology to *M*.*tb* SecA2. To evaluate whether *M*.*avium* also release their RNA into host cells and induce type I IFN production, we initially infected mouse bone marrow-derived macrophages (BMMs) with *M*.*avium* strains 104 and 2151. As shown in Fig 1A, *M*.*avium* significantly induced IFN-β production at 24 and 72 hr post-infection. An IFN-β mRNA expression peak was detected at 8 hr post-infection (Fig1B), which was similar to our finding with *M*.*tb* [5]. *M*.*avium* RNAs in the cytosol of infected macrophages were detected by quantitative RT-PCR. As seen in Fig 1C, four *M*.*avium* transcripts, *mce1B*, *polA*, *rpoC* and *MAV4436*, were detected in *M*.*avium*-infected but not uninfected macrophages. However, we did not detect 16S ribosomal RNA in the macrophage cytosol suggesting that the detected RNA was not due to contaminating lysed M.avium (Fig 1C). To determine the link between *M*.*avium* RNA and type I IFN production, we measured the IFN-β production in WT and *Mavs*^*–/–*^BMMs following an *M*.*avium* infection. As shown in Fig 1D and 1E, the *M*.*avium*-induced IFN-β production was significantly diminished in *Mavs*^*–/–*^relative to WT BMMs. Similar results were detected in *M*.*avium*-infected BMMs pretreated with RIG-I, TBK1, IRF3 or IRF7 siRNA when compared to cell pretreated with control siRNAs (Fig 1F and 1G, and S1 Fig). IRF3 and IRF7 are two critical transcription factors driving IFN-β expression in the context of viral and *M*.*tb* infection [3,5] and their activation and translocation into the nucleus of host cells requires phosphorylation by TBK1. As seen in Fig 1H, *M*.*avium* infection induced IRF3 and IRF7 nuclear translocation in WT but not *Mavs*^*–/–*^BMMs at 24 hr post-infection. As observed with an *M*.*tb* infection, IRF7 expression was induced by *M*.*avium* in WT BMMs. However, in contrast to *M*.*tb*, *M*.*avium* infection led to IRF3 nuclear translocation via a MAVS-dependent pathway. As predicted, MAVS was required for TBK1 phosphorylation in *M*. *avium*-infected BMMs (Fig 1I).

### The MAVS-dependent RNA sensing pathway is required for host defense against *M*.*avium* infection in mice

**Fig 2:**
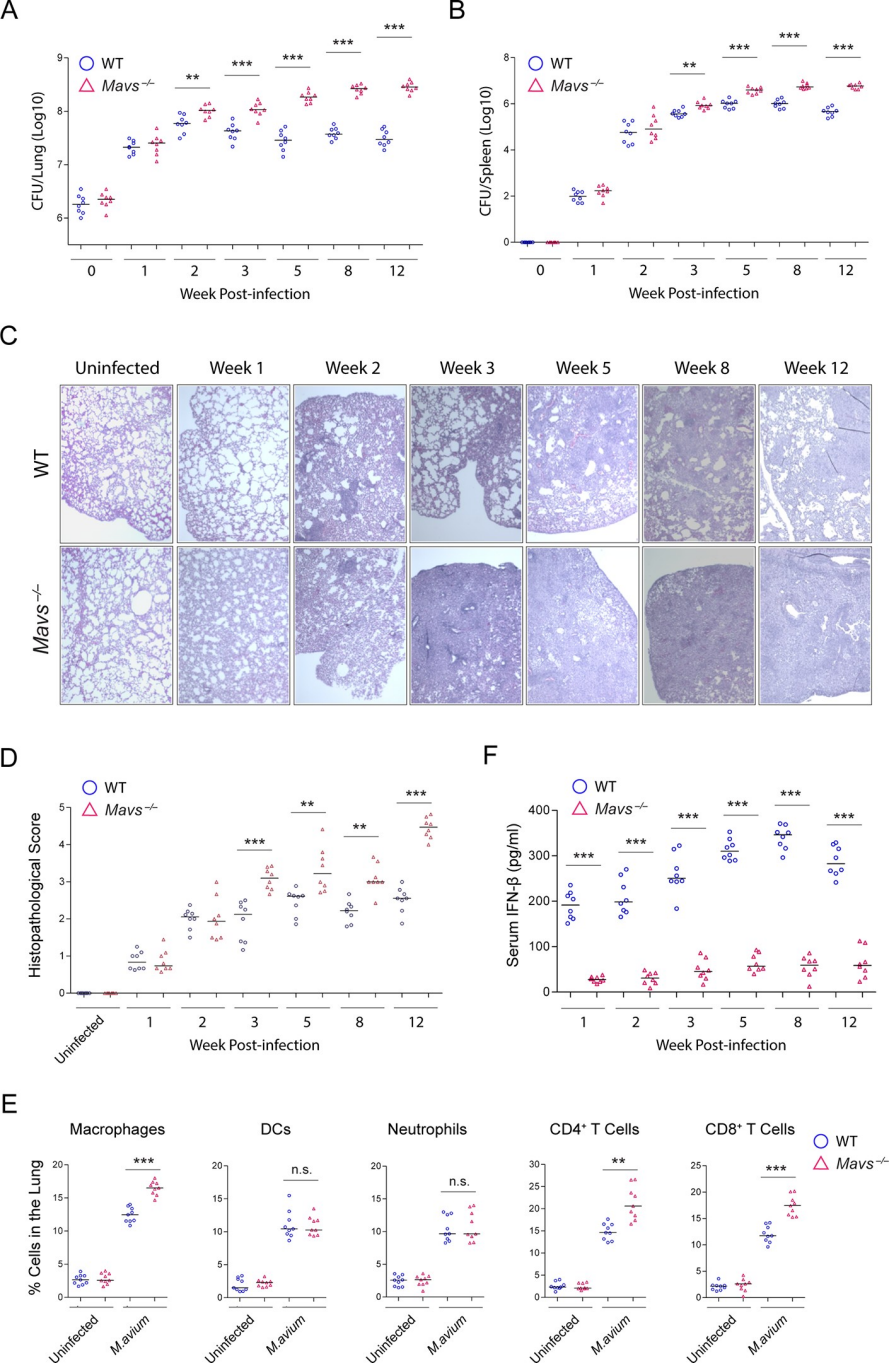
A MAVS-mediated RNA sensing pathway is required for *M*.*avium* control in mice. (A and B) *M*. *avium* 104 burden in the lung (A) and spleen (B) of WT and *Mavs*^*-/-*^ mice at various time points post *M*. *avium* intratracheal infection at a dose of 1x10^7^ CFU/mouse. (C) Representative H&E staining of lung sections from *M*.*avium*-infected WT and *Mavs*^*-/-*^ mice at various time points post infection. (D) Histopathological scoring of H&E stained lung sections at various time points post *M*.*avium* infection. (E) Flow cytometry analysis for the composition of the different immune cells in WT and *Mavs*^*-/-*^ mouse lungs at 3 weeks post *M*.*avium* infection. Shown as % of total cells counted. (F) ELISA analysis for IFN-β in serum of WT and *Mavs*^*-/-*^ mice at various time points post *M*.*avium* infection. All data shown are the combination of two (A-D) or three (E and F) independent experiments. n.s., not statistically significant; **P < 0.01 and ***P < 0.001 by Mann–Whitney U test.

To further evaluate the host cytosolic RNA sensing pathway we intratracheally infected WT and *Mavs*^*–/–*^C57BL/6 mice with wild-type *M*.*avium* and measured mycobacterial survival and tissue histopathology at 0, 1, 2, 3, 5, 8 and 12 weeks post-infection. As shown in Fig 2A, MAVS deficiency resulted in a 0.5 to 1.0 log_10_ increase in bacterial burden in the lung at 3, 5, 8 and 12 weeks post-infection when compared to WT infected mice. A similar difference in *M*.*avium* burden was observed in mouse spleen at 5, 8 and 12 weeks post-infection (Fig 2B). Consistent with *M*.*avium* survival results, the lungs from *M*.*avium*-infected *Mavs*^*–/–*^mice showed greater histopathological changes and immune cell infiltration at 3, 5, 8 and 12 weeks post-infection (Fig 2C and 2D). Flow cytometry analysis at 3 weeks post-infection showed the lungs of *M*.*avium*-infected *Mavs*^*–/–*^mice to have a higher concentration of macrophages and T cells when compared to *M*. *avium*-infected WT mice (Fig 2E). No difference in the number of DCs and neutrophils was detected in the lung between *M*.*avium*-infected WT and *Mavs*^*–/–*^mice. Similar to the results seen in BMMs, diminished IFN-β production was observed in the serum of *M*.*avium*-infected MAVS^-/-^ relative to infected WT mice (Fig 2F).

### RIG-I/MAVS/TBK1-dependent and IRF3/7-independent RNA sensing pathway regulates ICAM-1 Production in *M*.*avium*-infected host cells

**Fig 3:**
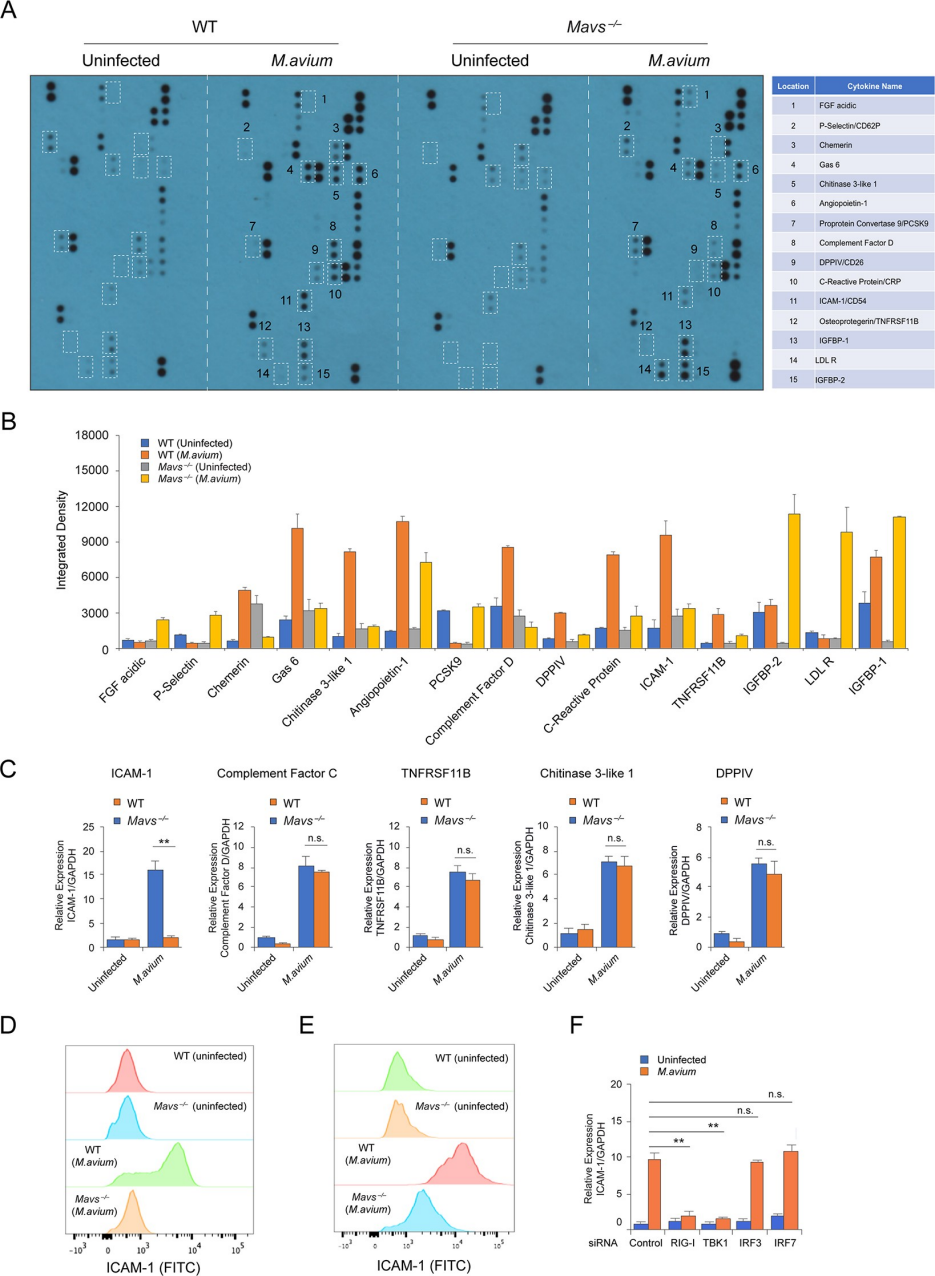
*M*. *avium* induces ICAM-1 production in macrophages through a RIG-I/MAVS/TBK1-dependent pathway. (A) Cytokine array analyzing indicated serum proteins in WT and *Mavs*^*-/-*^ mice 3 weeks post *M*. *avium* intratracheal infection using a dose of 1x10^7^ CFU/mouse. (B) Densitometry of serum proteins highlighted in (A). (C) qRT-PCR analysis of designated transcripts in uninfected or *M*. *avium* 104 infected WT and *Mavs*^*-/-*^ BMMs 24 hr post infection. Uninfected WT set to 1.0. (D) Flow cytometry analysis for ICAM-1 expression by uninfected or *M*. *avium* 104 infected WT and *Mavs*^*-/-*^ BMMs 24 hr post infection. (E) Flow cytometry analysis for ICAM-1 surface expression on AMs isolated from uninfected or *M*.*avium*-infected WT and *Mavs*^*-/-*^ mice 3 weeks post infection. (F) qRT-PCR for ICAM-1 in *M*.*avium*-infected WT BMMs (24 hr post infection) that were pretreated with control, RIG-I, TBK1, IRF3 or IRF7 siRNA. Control uninfected set to 1.0 Data shown in (C and F) are the mean ±SD (n = 3 wells per group), and all data are representative of two (A and B) or three (C-F) independent experiments. In qRT-PCR, GAPDH served as loading control and data was expressed as fold change relative to uninfected WT (C), or control uninfected (F) cells. n.s., not statistically significant and **P < 0.01 by Student’s t-test (two-tailed).

MAVS deficiency attenuated the host defense against an *M*.*avium* mouse infection. This result is in contrast to what we observed in *M*.*tb*-infected mice where *M*.*tb* burden was significantly lower in *Mavs*^*–/–*^infected mice when compared to WT [5]. To better understand the MAVS-dependent mycobacterial killing, we investigated the serum cytokine profile in WT and *Mavs*^*–/–*^mice post *M*.*avium* infection. As shown in Fig 3A and 3B, we identified 15 proteins that were differential produced in *M*.*avium*-infected WT and *Mavs*^*–/–*^mice at 3 weeks post-infection. The production of 6 proteins, IGFBP-1, IGFBP-2, LDL R, P-Selectin, PCSK9 and FGF acidic, increased in *Mavs*^*–/–*^vs. WT mice. Nine proteins, Angiopoietin-1, Chitinase 3-like 1, Chemerin, Complement Factor D, C-Reactive Protein, DPPIV, Gas6, ICAM-1 and TNFRSF11B, were downregulated in *Mavs*^*–/–*^vs. WT mice. As *M*.*avium* is an intracellular bacterial pathogen and primarily infects alveolar macrophages, we tested the expression of these 9 downregulated proteins in uninfected and *M*.*avium*-infected BMMs. Among those tested, we only detected 5 mRNA (Chitinase 3-like 1, Complement Factor D, DPPIV, ICAM-1 and TNFRSF11B) by qRT-PCR, and all showed an *M*.*avium*-inducible expression in WT BMMs. ICAM-1 was the only protein whose expression was decreased in *Mavs*^*–/–*^BMMs compared to WT cells (Fig 3C), ICAM-1 is a cell surface glycoprotein expressed on endothelial cells and immune cells but can also be detected extracellularly [14]. Based on the qRT-PCR results we analyzed the abundance of surface ICAM-1 on macrophages using flow cytometry. As seen in Fig 3D, compared to uninfected BMMs, *M*.*avium* infection significantly increased ICAM-1 surface expression in WT but not *Mavs*^*–/–*^BMMs. A similar result was observed in alveolar macrophages isolated from WT and *Mavs*^*–/–*^mice at 3 weeks post *M*. *avium* infection (Fig 3E). In contrast, a comparable level of ICAM-1 was detected in DCs, neutrophils, CD4^+^ and CD8^+^ T cells in the lung of *M*. *avium*-infected WT and *Mavs*^*–/–*^mice (S2A Fig). Interestingly, RIG-I-/TBK1- but not IRF3/7-knockdown diminished ICAM-1 production in *M*.*avium*-infected BMMs (Fig 3F), suggesting an undefined downstream pathway mediating ICAM-1 production in host cells.

### RIG-I/MAVS/TBK1-dependent RNA sensing pathway mediates ETV5 stability in BMMs

**Fig 4:**
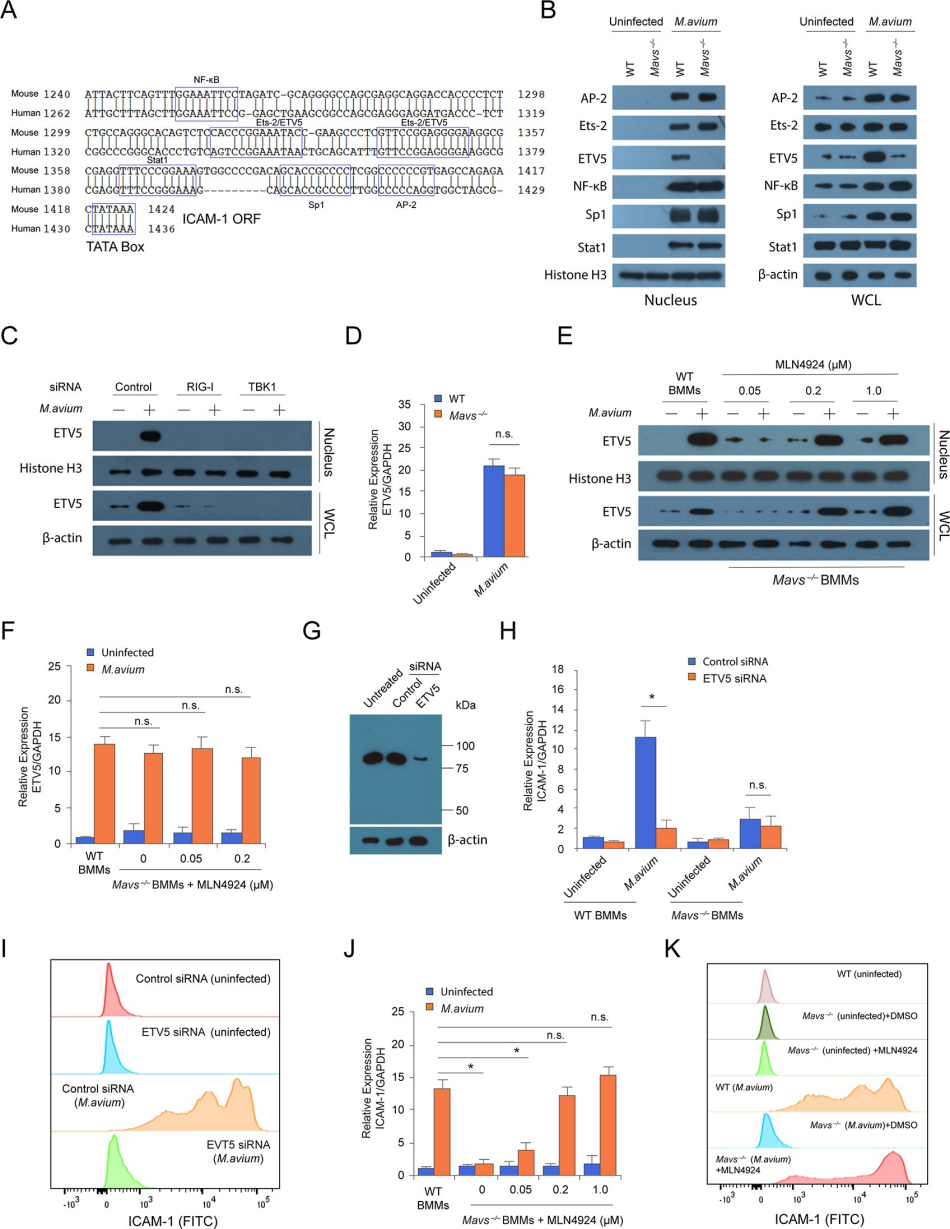
Increased ETV5 protein stability through RIG-I/MAVS/TBK1 activation is required for *M*. *avium*-induced ICAM-1 expression in macrophages. (A) Transcription factors and recognition sites on the promoter of mouse and human ICAM-1 gene. (B) Western blot analysis for transcription factors (A) in the nuclear fraction (Nucleus) and WCL of WT and *Mavs*^*-/-*^ BMMs uninfected or infected with *M*.*avium* for 24 hr. (C) Western blot analysis for ETV5 in BMMs 24 hr after *M*.*avium*-infection in cells that were pretreated with control, RIG-I or TBK1 siRNA. (D) qRT-PCR for ETV5 in WT and *Mavs*^*-/-*^ BMMs uninfected or infected with *M*.*avium* for 24 hr. Uninfected WT set to 1.0. (E) Western blot for ETV5 in nuclear fraction (Nucleus) and WCL of *Mavs*^*-/-*^ BMMs infected with *M*.*avium* for 24 hr and treated with different concentrations of MLN4924 at. (F) qRT-PCR for ETV5 in *Mavs*^*-/-*^ BMMs following a 24 hr infection with *M*. *avium* 104 treated with MLN4924 at various concentrations. Uninfected WT set to 1.0. (G) Western blot analysis for ETV5 in BMMs infected for 24 hr with M. avium 104. Cells were pretreated with negative control or EVT5 siRNA for 48 hr prior to infection. (H) qRT-PCR for ICAM-1 production in WT and *Mavs*^*-/-*^ BMMs pretreated with control or ETV5 siRNA, and then left uninfected or infected with *M*.*avium* for 24 hr. Uninfected WT set to 1.0. (I) Similar to (H), but flow cytometry analysis for ICAM-1. (J) Similar to (F), but qRT-PCR for ICAM-1. (K) Flow cytometry analysis for ICAM-1 in *Mavs*^*-/-*^ BMMs infected with *M*.*avium* for 24 hr in the presence of MLN4924 (0.5 μM). Data shown in (D, F, H and J) are the mean ±SD (n = 3 wells per group), and all data are representative of three independent experiments. In qRT-PCR, GAPDH served as loading control and data was expressed as fold change relative to uninfected WT cells or control siRNA-treated uninfected WT cells. n.s., not statistically significant and *P < 0.05 by Student’s t-test (two-tailed).

As MAVS-dependent production of ICAM-1 in *M*. *avium*-infected BMM did not require IRF3 or IRF7 we analyzed the promoter region in both human and mouse ICAM-1 gene to identify conserved transcription factor-binding sites (Fig 4A), We found NF-κB, Ets-2, ETV5, Ap-2, Sp-1 and Stat1 binding sites [15] and hypothesized that MAVS deficiency alters the activation of one or more of these factors induced upon an *M*.*avium* infection. To test this hypothesis we investigated the nuclear translocation of these 6 transcription factors in WT and *Mavs*^*–/–*^BMMs following an *M*.*avium* infection. Only ETV5 nuclear levels were significantly lower in *Mavs*^*–/–*^compared to WT BMMs (Fig 4B). A similar result was detected in the WCL. Moreover, ETV5 abundance in the nucleus and WCL was also diminished in *M*. *avium*-infected BMMs that were pretreated with RIG-I- or TBK1-specific siRNA relative to cells receiving control siRNA (Fig 4C). However, qRT-PCR analysis showed a comparable level of ETV5 mRNA in *M*. *avium*-infected WT and *Mavs*^*–/–*^BMMs (Fig 4D), suggesting limited ETV5 translation or diminished protein stability in *Mavs*^*–/–*^BMMs. Recently, it was found that ETV5 can be degraded through the cullin-RING ubiquitin ligase CRL4^COP1/DET1^-meidated ubiquitination in mouse alveolar type II cells [16]. This degradation was shown to be inhibited by MLN4924, an inhibitor of CRL4^COP1/DET1^ complex [17]. To test whether CRL4^COP1/DET1^-meidated ubiquitination was involved in the diminished ETV5 expression observed in *M*.*avium*-infected *Mavs*^*–/–*^BMMs, we inactivated the CRL4^COP1/DET1^ enzyme complex using MLN4924. As shown in Fig 4E, inhibiting CRL4^COP1/DET1^ rescued ETV5 protein production in *Mavs*^*–/–*^BMMs while having no effect on EVT5 mRNA abundance (Fig 4F).

To further define ETV5’s role in *M*.*avium*-induced ICAM-1 expression, we evaluated the ICAM-1 production in WT BMMs pretreated with ETV5-specific siRNA. As seen in Fig 4G, 4H and 4I EVT5 knockdown significantly decreased ICAM-1 expression at both the mRNA (Fig 4H) and protein (Fig 4I) level in *M*.*avium*-infected WT BMMs. Moreover, treatment with MLN4924 lead to recovered ICAM-1 RNA and protein production in *Mavs*^*–/–*^BMMs following infection with *M*.*avium* (Fig 4J and 4K).

### MAVS deficiency attenuates CD4^+^ T Cell-mediated mycobacterial killing in BMMs

**Fig 5:**
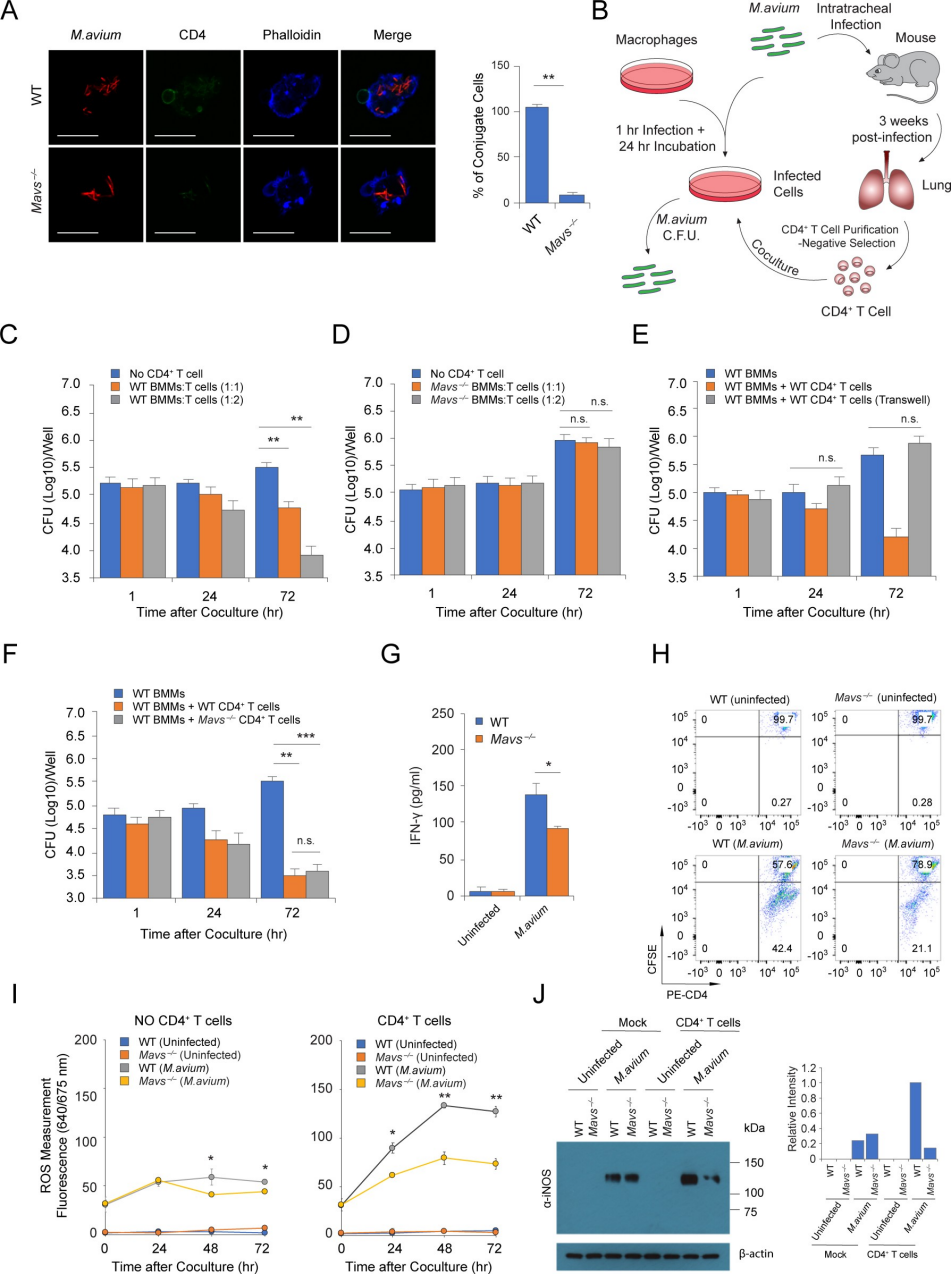
MAVS-dependent RNA sensing pathway contributes to CD4^+^ T cell-mediated mycobacterial killing in macrophages. (A) Confocal microscopy analysis of *M*. *avium* (tdTomato)-infected BMMs and CD4 T cells (Alexa Fluor 488) following a 24 hr coculture. Scale bar, 20 μm. For quantification, all data was normalized to one biological experiment using WT BMMs, which was set to 100%. (B) Schematic of macrophage-T cell coculture assay. (C) *M*. *avium* burden in WT infected BMMs cocultured with CD4^+^ T cells isolated from *M*.*avium*-infected WT mice at a cell ratio (BMMs:T cells) of 1:1 or 1:2. (D) *M*. *avium* burden in *Mavs*^*-/-*^ infected BMMs cocultured with CD4^+^ T cells isolated from *M*. *avium*-infected WT mice at a cell ratio (BMMs:T cells) of 1:1 or 1:2. (E) *M*. *avium* burden in WT infected BMMs (Bottom Well) cocultured directly with CD4^+^ T cells or with CD4^+^ T cells in the upper chamber of a transwell. CD4^+^ T cells were isolated from WT mice 3 weeks post *M*. *avium* infection. (F) *M*. *avium* burden in WT infected BMMs cocultured with CD4^+^ T cells isolated from WT or *Mavs*^*-/-*^ mice 3 weeks post *M*. *avium* infection. (G) IFN-γ levels in the culture supernatant of uninfected or *M*. *avium*-infected WT and *Mavs*^*-/-*^ BMMs cocultured for 72 hr with CD4^+^ T cells isolated from *M*. *avium*-infected WT mice. (H) Flow cytometry analysis of CFSE stained CD4^+^ T cells isolated from *M*. *avium*-infected WT mice that were cocultured for 72 hr with *M*. *avium*-infected WT or *Mavs*^*-/-*^ BMMs. (I) ROS measurement in *M*. *avium*-infected WT and *Mavs*^*-/-*^ BMMs +/- cocultured with CD4^+^ T cells isolated from WT mice 3 weeks post *M*. *avium* infection. (J) Western blot for iNOS expression in *M*. *avium*-infected WT and *Mavs*^*-/-*^ BMMs +/- coculture for 24 hr with CD4^+^ T cells isolated from WT mice 3 weeks post *M*. *avium* infection. Quantitation of band pixel intensity shown in graph. Data shown in (C-G) and (I) are the mean ±SD (n = 3 independent infections per group), and all data are representative of at least three independent experiments. In (E-G), the ratio between BMMs and CD4^+^ T cells was 1:2. n.s., not statistically significant, *P < 0.05, **P < 0.01 and ***P < 0.001 by Student’s t-test (two-tailed).

To understand the role of host cytosolic RNA sensing pathway in the antimycobacterial response, we investigated *M*.*avium* replication in *Mavs*^*–/–*^BMMs and RIG-I, TBK1, IRF3 or IRF7-knockdown BMMs. As seen in S3A and S3B Fig, a minimal increase in mycobacterial burden was detected in *Mavs*^*–/–*^BMMs as well as RIG-I, TBK1, IRF3 or IRF7-knockdown BMMs when compared to WT or control siRNA-treated cells at 72 hr post infection. This suggest that the host cytosolic RNA sensing pathway plays a limited role in the antimycobacterial response. In addition to its role as an adhesion molecule for cell-cell interaction, ICAM-1 is also an essential component of the immunological synapse between antigen-presentation cells and activated lymphocytes [9]. CD4^+^ T cell-mediated cellular immune response plays a critical role in controlling mycobacterial infections [18]. Therefore, we hypothesized that MAVS-dependent ICAM-1 production in *M*.*avium*-infected macrophages could facilitated CD4^+^ T cell-mediated mycobacterial killing in host cells. To test our hypothesis, we isolated CD4^+^ T cells from *M*.*avium* infected mice and cocultured these T cells with *M*.*avium*-infected BMMs. As seen in Fig 5A, we observed significantly fewer CD4^+^ T cells associated with *M*.*avium*-infected *Mavs*^*–/–*^compared to WT infected BMMs. To determine if this difference in T cell-BMM interaction results in changes in *M*.*avium* survival, we lysed the infected BMMs following coculture and quantified the bacterial burden (Fig 5B). As shown in Fig 5C, *M*.*avium* burden declined by ~1.5 log10 in WT BMMs cocultured with CD4^+^ T cells (cell ratio, 1:2) isolated from *M*.*avium*-infected WT mice compared to BMMs alone. In contrast, CD4^+^ T cell-mediated mycobacterial killing was absent when these CD4^+^ T cells were cocultured with *M*.*avium*-infected *Mavs*^*–/–*^cells (Fig 5D). Limited effect on *M*.*avium* survival was observed when CD8^+^ T cell isolated from *M*.*avium*-infected WT mice, were added to *M*.*avium* infected WT BMMs (S3C Fig). CD4^+^ or CD8^+^ T cells from naïve WT mice had no effect on *M*.*avium* survival in host cells (S3D Fig). To investigate whether CD4^+^ T cell-mediated mycobacterial killing relies on cell-cell contact, we assessed *M*. *avium* burden in BMMs cocultured with CD4^+^ T cells from *M*. *avium*-infected WT mice in a transwell system. As shown in Fig 5E, CD4^+^ T cells failed to activate antimycobacterial response in the absence of cell-cell contact. To better understand the role of the cytosolic RNA sensing pathway in host defense against *M*.*avium* infection *in vivo*, we compared the capability of CD4^+^ T cells isolated from *M*.*avium*-infected WT and *Mavs*^*–/–*^mice at 3 weeks post infection to stimulate mycobacterial killing when cocultured with *M*.*avium*-infected WT BMMs. As seen in Fig 5F, CD4^+^ T cells from the two mouse strains had similar antimicrobial activity. In parallel, a comparable level of IFN-γ was detected in CD4^+^ and CD8^+^ T cells isolated from *M*. *avium*-infected WT and *Mavs*^*–/–*^mouse lung (S2B and S2C Fig). This suggest that MAVS is not required for activation of Th_1_ CD4^+^ T cell in *M*.*avium*-infected mice. In contrast, using the macrophage-CD4^+^ T cell coculture system we found that MAVS deficiency in host macrophages attenuated IFN-γ production by the CD4^+^ T cells (Fig 5G). Similarly, less T cell proliferation was detected when CD4^+^ T cells, isolated from infected mice, were cocultured with *M*.*avium*-infected *Mavs*^*–/–*^cells compared to CD4^+^ T cells cocultured with infected WT cells (Fig 5H).

Following coculture with activated antigen-specific CD4^+^ T cells, *M*.*tb*-infected macrophages are known to produce antimicrobial reactive oxygen and nitrogen species (ROS and RNS) via NADPH oxidase (NOX2/gp91phox) and inducible nitric oxide synthase (iNOS) respectively [19]. However, little is known about ROS and NO production in the context of NTM infections. Since MAVS deficiency in *M*.*avium* infected BMMs resulted in a loss of CD4^+^ T cell-mediated killing of *M*.*avium*, we investigated if the production of ROS and RNS was altered in *M*.*avium*-infected *Mavs*^*–/–*^compared to WT BMMs following cocultured with CD4^+^ T cells isolated from *M*.*avium*-infected WT mice. In the presence of CD4^+^ T cells, the level of ROS (Fig 5I) and iNOS (Fig 5J) was significantly higher in WT compared to *Mavs*^*–/–*^*M*.*avium*-infected BMMs. However, no significant difference in ROS and iNOS production was detected between *M*.*avium*-infected WT and *Mavs*^*–/–*^BMMs in the absence of CD4^+^ T cells.

### MAVS deficiency attenuates CD4^+^ T Cell-mediated mycobacterial killing in mice

**Fig 6:**
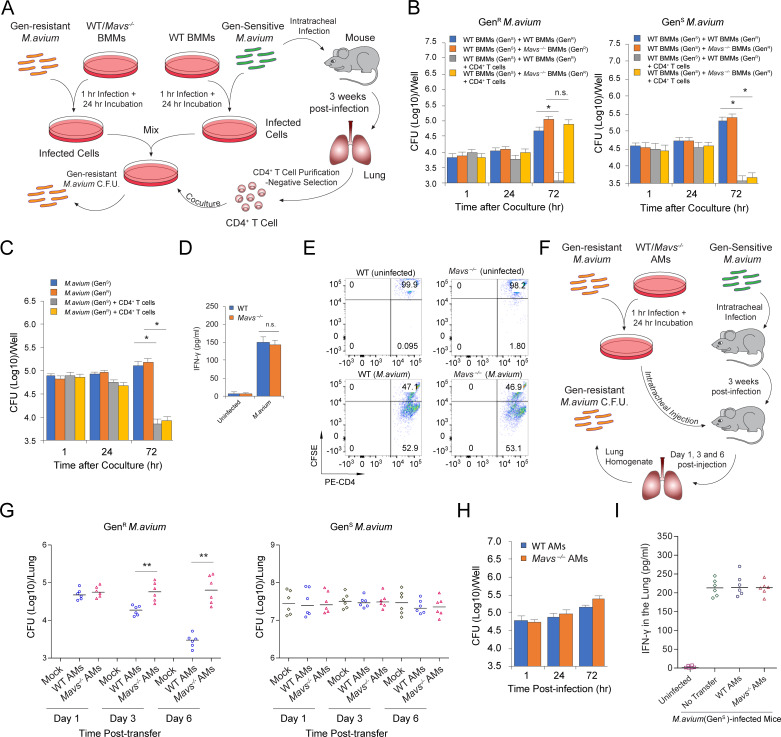
MAVS-dependent RNA sensing pathway contributes to CD4^+^ T cell-mediated mycobacterial killing in mice. (A) Schematic of coculture assay with triple cell populations. (B) Gentamycin-resistant (Gen^R^) and -sensitive (Gen^S^) *M*.*avium* burden in WT and *Mavs*^*-/-*^ BMMs cocultured with CD4^+^ T cells from *M*.*avium*-infected WT mice using the triple-cell coculture system. (C) Bacterial counts of Gen^R^ and Gen^S^ *M*.*avium* strains in WT BMMs +/- coculture with CD4^+^ T cells purified from *M*.*avium*-infected WT mice. (D) IFN-γ concentration in the culture supernatant after 72 hr incubation of the triple-cell coculture system as described in (B). (E) Similar to (D), but flow cytometry analysis for CFSE stained CD4^+^ T cells following a 72 hr coculture with CD4^+^ T cells. (F) Schematic of the adoptive transfer assay using *M*.*avium*-infected AM and WT mice. (G) Gen^R^ and Gen^S^ *M*.*avium* burden in WT mouse lung at day 1, 3 and 6 after *M*.*avium*-infected AM adoptive transfer. WT and *Mavs*^*-/-*^ AM were isolated from WT and *Mavs*^*-/-*^ naïve mice, respectively. Mock, no injection. (H) Gen^R^ *M*.*avium* burden in WT and *Mavs*^*-/-*^ AMs in vitro. (I) IFN-γ concentration in WT mouse lungs 6 days after the *M*.*avium*-infected AM transfer. Data in (B-D) and (H) are the mean ±SD (n = 3 independent infections per group), and data shown are representative of at least three (B-E, and H) or combination of two (G and I, n = 3 per group each experiment) independent experiments. In triple-cell population coculture, a ratio: Gen^S^ *M*.*avium*-infected BMMs: Gen^R^ *M*.*avium*-infected BMMs:T cells = 4:1:10, was used. n.s., not statistically significant, *P < 0.05 and **P < 0.01 by Student’s t-test (two-tailed) (B-D) or Mann–Whitney U test (G).

Our data indicates that MAVS deficiency impairs macrophage-T cell immune synapse formation upon in vitro coculture. However, a comparable level of IFN-γ was observed in CD4^+^ T cells (S2B Fig) and the lungs (S2C Fig) of *M*. *avium* infected WT and MAVS^-/-^ mice. We hypothesized that MAVS play a key role in *CD4+ T cell-mediated stimulation of macrophages to kill intracellular* M.avium but not CD4^+^ T cell activation in vivo. To test our hypothesis, we established a triple-cell population coculture system (Fig 6A), in which CD4^+^ T cells were isolated from WT mice at 3 weeks post *M*.*avium* infection. These T cells were then cocultured with WT BMMs infected with gentamicin-sensitive (Gen^S^) *M*.*avium*, followed by coculture with WT or *Mavs*^*–/–*^BMMs that were infected with gentamicin-resistant (Gen^R^) *M*.*avium* strains. The Gen^R^ and Gen^S^ bacterial burden in the BMMs were quantified. A difference in Gen^R^ *M*.*avium* burden between WT and *Mavs*^*–/–*^BMMs in coculture would indicate a role for MAVS in *CD4+ T cell-mediated stimulation of macrophages to kill intracellular* M.avium. As shown in Fig 6B, in the presence of CD4^+^ T cells isolated from *M*.*avium*-infected WT mice, Gen^R^ *M*.*avium* burden in WT BMMs was significantly lower compared to *Mavs*^*–/–*^BMMs. In contrast, in the absence of CD4^+^ T cells, Gen^R^ *M*.*avium* had a similar survival rate in WT and *Mavs*^*–/–*^BMMs. Similarly, Gen^S^ and Gen^R^ *M*.*avium* showed a comparable survival in WT BMMs (Fig 6C). CD4^+^ T cells from *M*.*avium*-infected WT mice did kill Gen^S^ *M*.*avium* in WT BMMs (Fig 6C). In the triple-cell coculture wells, a similar level of IFN-γ and T cell proliferation was seen regardless of whether Gen^R^ *M*.*avium*-infected WT or *Mavs*^*–/–*^BMMs were present, suggesting an effective activation of CD4^+^ T cells by Gen^S^ *M*.*avium*-infected WT BMMs (Fig 6D and 6E).

Our results indicate an important role for the host cytosolic RNA sensing pathway in the CD4^+^ T cell-mediated antimycobacterial response. To evaluate the engagement of MAVS in *M*.*avium*-infected macrophages in vivo, we performed an alveolar macrophage (AM) adoptive transfer experiment where WT or *Mavs*^*–/–*^AMs, which were infected with Gen^R^ *M*.*avium*, were intratracheally administered to WT mice that had been previously infected with the Gen^S^ *M*.*avium* for 3 weeks as described in Fig 6F. Gen^R^ *M*.*avium* burden in mouse lung was analyzed at day 1, 3 and 6 after intratracheal injection. Gen^R^ *M*.*avium* burden declined when the infected WT but not *Mavs*^*–/–*^AMs were injected into mice previously infected with Gen^S^ *M*.*avium* (Fig 6G). AM adoptive transfer had no effect on the Gen^S^ *M*.*avium* burden in mouse lung. In macrophage culture, Gen^R^ *M*. *avium* had a comparable survival rate in WT and *Mavs*^*–/–*^AMs (Fig 6H). Additionally, neither the transfer of WT or *Mavs*^*–/–*^AMs affected the IFN-γ production in the lung of Gen^S^ *M*.*avium*-infected WT mice (Fig 6I). WT and *Mavs*^*–/–*^AMs infected with Gen^R^ *M*.*avium* showed similar survival rate in Gen^S^ *M*.*avium*-infected WT mice over a 6-day period (S4A Fig).

### *M*.*avium*-induced ICAM-1 production contributes to CD4^+^ T Cell-mediated mycobacterial killing in BMMs and mice

**Fig 7:**
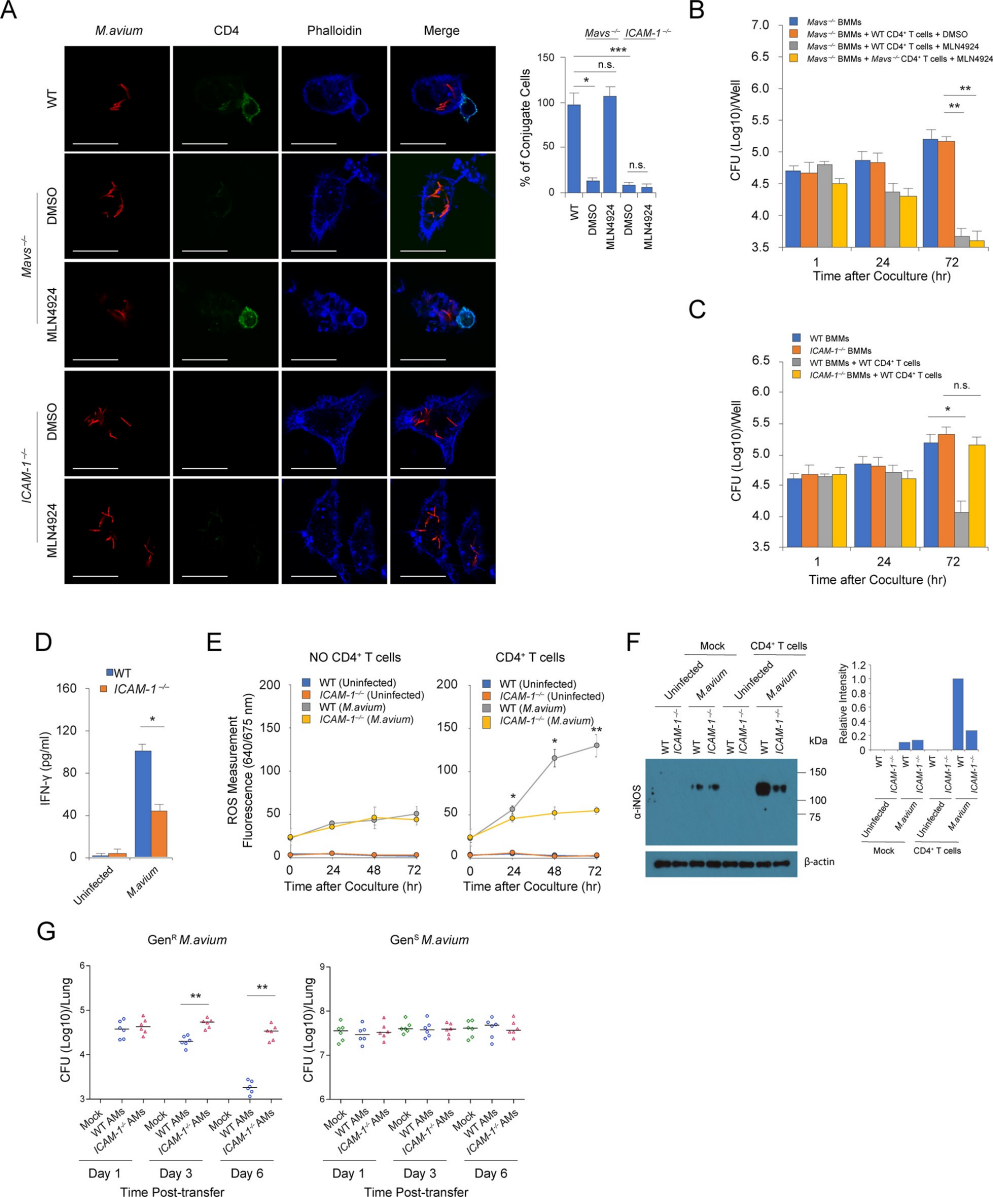
ICAM-1 production in host cells is essential for CD4^+^ T cell-mediated *M*. *avium* killing in macrophage and mice. (A) Confocal microscopy measuring the cell-cell interaction between *M*. *avium* (tdTomato)-infected WT, *Mavs*^*-/-*^ or *ICAM-1*^*-/-*^ BMMs and CD4^+^ T cells (Alexa Fluor 488) isolated from *M*. *avium*-infected WT mice +/- MLN4924 (0.5 μM). Images were taken 24 hr after BMM and CD4^+^ T cell coculture. Scale bar, 20 μm. For quantification, all data was normalized to one biological repeat in WT group. (B) *M*. *avium* burden in *Mavs*^*-/-*^ BMMs pretreated with MLN4924 (0.5 μΜ) and then cocultured with CD4^+^ T cells isolated from *M*. *avium*-infected WT or *Mavs*^*-/-*^ mice. (C) *M*.*avium* burden in WT and *ICAM-1*^*-/-*^ BMMs cocultured with CD4^+^ T cells isolated from *M*.*avium*-infected WT mice. (D) IFN-γ concentration in the culture supernatant of uninfected or *M*. *avium*-infected WT and *ICAM-1*^*-/-*^ BMMs cocultured for 72 hr with CD4^+^ T cells isolated from *M*. *avium*-infected WT mice. (E) ROS measured over time in *M*. *avium*-infected WT and *ICAM-1*^*-/-*^ BMMs +/- coculture with CD4^+^ T cells isolated from *M*. *avium*-infected WT mice. (F) Western blot for iNOS in *M*.*avium*-infected WT and *ICAM-1*^*-/-*^ BMMs +/- coculture for 24 hr with CD4^+^ T cells isolated from *M*. *avium*-infected WT mice. Quantitation of band pixel intensity shown in graph. (G) Gen^R^ and Gen^S^ *M*.*avium* burden in WT mouse lung at day 1, 3 and 6 after *M*.*avium*-infected AM adoptive transfer. WT AM and *ICAM-1*^*-/-*^ AMs were isolated from WT and *ICAM-1*^*-/-*^ naïve mice, respectively. Data in (A-E) are the mean ±SD (n = 3 independent infections per group), and data shown are representative of at least three (A-F) or combination of two (G, n = 3 per group each experiment) independent experiments. The ratio between BMMs and CD4^+^ T cells was 1:2. n.s., not statistically significant, *P < 0.05 and **P < 0.01 by Student’s t-test (two-tailed) (A-E) or Mann–Whitney U test (G).

Since ICAM-1 is necessary for the formation of immune synapse between APC and T cells^9^, we hypothesized that the impaired mycobacterial killing in *Mavs*^*–/–*^BMMs was due to diminished ICAM-1 production in infected host cells. To test this hypothesis we first defined the surface expression of additional proteins required for the APC-T cell synapse in *M*.*avium*-infected BMMs. MHCII, CD80, CD86 and CD40 showed similar abundance on the plasma membrane of *M*.*avium*-infected WT and *Mavs*^*–/–*^BMMs (S2D Fig and Fig 3D). To further test our hypothesis, we infected WT and *Mavs*^*–/–*^BMMs with *M*.*avium* in the presence of MLN4924 before cells were cocultured with CD4^+^ T cells isolated from *M*.*avium*-infected WT mice. To avoid the potential interference of MLN4924 on CD4^+^ T cells, MLN4924 was not included in macrophage-T cell coculture system. MLN4924 restored the immune synapsis between CD4^+^ T cells and *M*.*avium*-infected *Mavs*^*–/–*^BMMs but had no effect on immune synapsis formation when using *ICAM-1*^*–/–*^BMMs (Fig 7A). MLN4924 also rescued the antimycobacterial activity in *M*.*avium*-infected *Mavs*^*–/–*^BMMs cocultured with CD4^+^ T cells to levels comparable to what was observed in *M*.*avium*-infected WT BMMs (Fig 7B). As predicted, CD4^+^ T cell failed to control *M*.*avium* infection in *ICAM-1*^*–/–*^BMMs in comparison with *M*.*avium*-infected WT BMMs (Fig 7C). Consistent with MAVS deficiency, loss of ICAM-1 expression on infected BMMs resulted in attenuated CD4^+^ T cell activation characterized as defined by decreased IFN-γ production (Fig 7D). In addition, loss of ICAM-1 expression also impaired ROS (Fig 7E) and iNOS (Fig 7F) production by *M*.*avium*-infected BMMs following coculture with antigen-specific and activated CD4^+^ T cells. Finally, *ICAM-1*^*–/–*^AMs also showed limited control of a *M*. *avium* infection compared to WT AMs when cells were transferred to *M*. *avium*-infected WT mice (Fig 7G and S4B Fig).

## Discussion

Our previous study demonstrated that *M*.*tb* releases mycobacterial RNAs into the cytosol of host cells and stimulates the cytosolic RIG-1/MAVS/TBK1/IRF7 RNA sensing pathway and production of type I IFNs^5^. A similar result has been observed in *L*. *monocytogenes*[4]. In this study, we demonstrate that *M*.*avium*, like *M*.*tb*, activates the host cytosolic RIG-I/MAVS/TBK1/IRF3/IRF7 RNA sensing pathway and stimulate type I IFN production in infected macrophages. This extends to *in vivo* as MAVS deficiency attenuates IFN-β production in mice post *M*.*tb* or *M*.*avium* infection. However, in contrast to *M*.*tb*, *M*.*avium* burden was higher in *Mavs*^*–/–*^relative to WT mice and showed increased immunopathology during the course of the *M*.*avium* infection. This differential response to the MAVS defect following an *M*.*tb* and *M*.*avium* infection suggests that the RNA sensing pathway play significantly different roles in controlling these two mycobacterial infections in mice. We also found that the cytosolic RIG-I/MAVS/TBK1 but not IRF3/IRF7 RNA sensing pathway regulates ICAM-1 production in macrophages by inhibiting CRL4^COP1/DET1^-mediated ETV5 degradation in *M*. *avium*-infected cells. Although the ICAM-I production is controlled by multiple transcription factors including NF-κB, Ets-2, ETV5, Ap-2, Sp-1 and Stat1 [15], our data indicates that ETV5 is a key transcription factor in *M*. *avium*-induced ICAM-1 expression in macrophages. The ICAM-1 production in *M*. *avium*-infected *Mavs*^*–/–*^macrophages was restored by inhibition of the CRL4^COP1/DET1^ enzyme complex.

The in vitro macrophage killing assay reveals that the RIG-I/MAVS/TBK1 RNA sensing pathway plays a limited role in controlling *M*.*avium* infection in BMMs. However, the mouse infection studies suggest that this pathway may be playing a larger role *in vivo*. CD4^+^ T cells are essential in protective immunity against *M*.*tb* and *M*.*avium* infection in animal models and patients^18^ and their recruitment and/or activation may be dysregulated in the absence of RNA cytosolic RNA sensing pathway. Our findings that ICAM-1 expression is diminished in BMMs that lack RIG-I or MAVS expression following an *M*.*avium* infection suggest a link between RNA sensing pathways and an immune response to this bacterial infection. Multiple studies have shown ICAM-1 production is induced in host cells following various bacterial infections including *M*.*tb*, *M*.*avium*, *S*. *pneumoniae* and *Salmonella* [20–24], although the pathways leading to its expression were not defined in these studies. While the role of ICAM-1 during bacterial infections has been investigated using *ICAM-1*^*-/-*^ mice, little is known about ICAM-1’s function in promoting an antibacterial response within infected host cells [11,23–26]. The engagement of ICAM-1 in APC-T cell interaction prompted us to test whether the host cytosolic RIG-I/MAVS/TBK1 RNA sensing pathway facilitates CD4^+^ T cell-mediated mycobacterial killing during an *M*.*avium* infection. Interestingly, *M*.*avium*-infected *Mavs*^*–/–*^BMMs fail to form immune synapse with CD4^+^ T cells isolated from *M*.*avium*-infected WT mice. Moreover, *M*.*avium* burden was significantly higher in *Mavs*^*–/–*^*vs* WT macrophages cocultured with these CD4^+^ T cells. This defect in *M*.*avium* killing by *Mavs*^*–/–*^macrophages was restored by stimulating ICAM-1 production through the inhibition of CRL4^COP1/DET1^. Consistent with the *in vitro* macrophage killing study, the adoptive transfer experiment using *Mavs*^*–/–*^and *ICAM-1*^*–/–*^AMs supports the importance of MAVS-dependent pathway and ICAM-1 in APC-T cell interaction during a mouse *M*.*avium* infection. While CD8^+^ T cells are also important in the immune response against *M*.*tb* infection, it may play a lesser role in immunity to *M*.*avium* at least in the context of a mouse infection [27]. Our results support this finding as CD8^+^ T cells isolated from *M*.*avium*-infected WT mice showed limited ability to kill *M*. *avium* when added to infected macrophages in a coculture system. Interestingly, except for ICAM-1, MAVS deficiency has no measurable effect on the cell surface expression of various other molecules involved in APC-CD4^+^ T cell synapse formation including MHC II, CD80, CD86 and CD40.

In the context of an NTM infection, the regulation of the various molecules that mediate adhesion between Ag-specific CD4^+^ T cells and infected macrophages remains poorly defined. In this study we observed that the RIG-I/MAVS/TBK1/ETV5/ICAM-1 macrophage response to a *M*. *avium* infection significantly increases the CD4^+^ T cell-mediated activation and the antimycobacterial response. Our data suggest that this MAVS-dependent antimicrobial response is not mediated by IFN-γ. Evidence to support this conclusion includes: 1) When cocultured with CD4^+^ T cells, the MAVS/ICAM-1-dependent control of the *M*. *avium* infection was only partially mimicked by blocking IFN-γ following incubation of activated CD4+ with infected BMM. 2) A similar percentage of IFN-γ-producing CD4^+^ T cells and a comparable level of IFN-γ production was detected in the lung of *M*. *avium*-infected WT and *Mavs*^*–/–*^mice. 3) CD4^+^ T cells isolated from *M*. *avium*-infected *Mavs*^*–/–*^and WT mice have a comparable activity in stimulating antimycobacterial activity when added to *M*. *avium*-infected WT BMMs. In addition, a similar *M*. *avium* burden was detected in WT AMs that were transferred into WT and *Mavs*^*–/–*^mice. Altogether, our data indicates that there is a cell-cell contact dependent mechanism by which CD4^+^ T cells promote *M*. *avium* killing in host cells. Mechanisms for the T cell mediated response could include macrophage activation by the CD40-CD40L axis and downstream effects such as iNOS expression and NO production [28,29]. However, we cannot completely rule out that the MAVS^-/-^ and ICAM^-/-^ infected macrophages may also be more refectory to cytokine activation.

**Fig 8:**
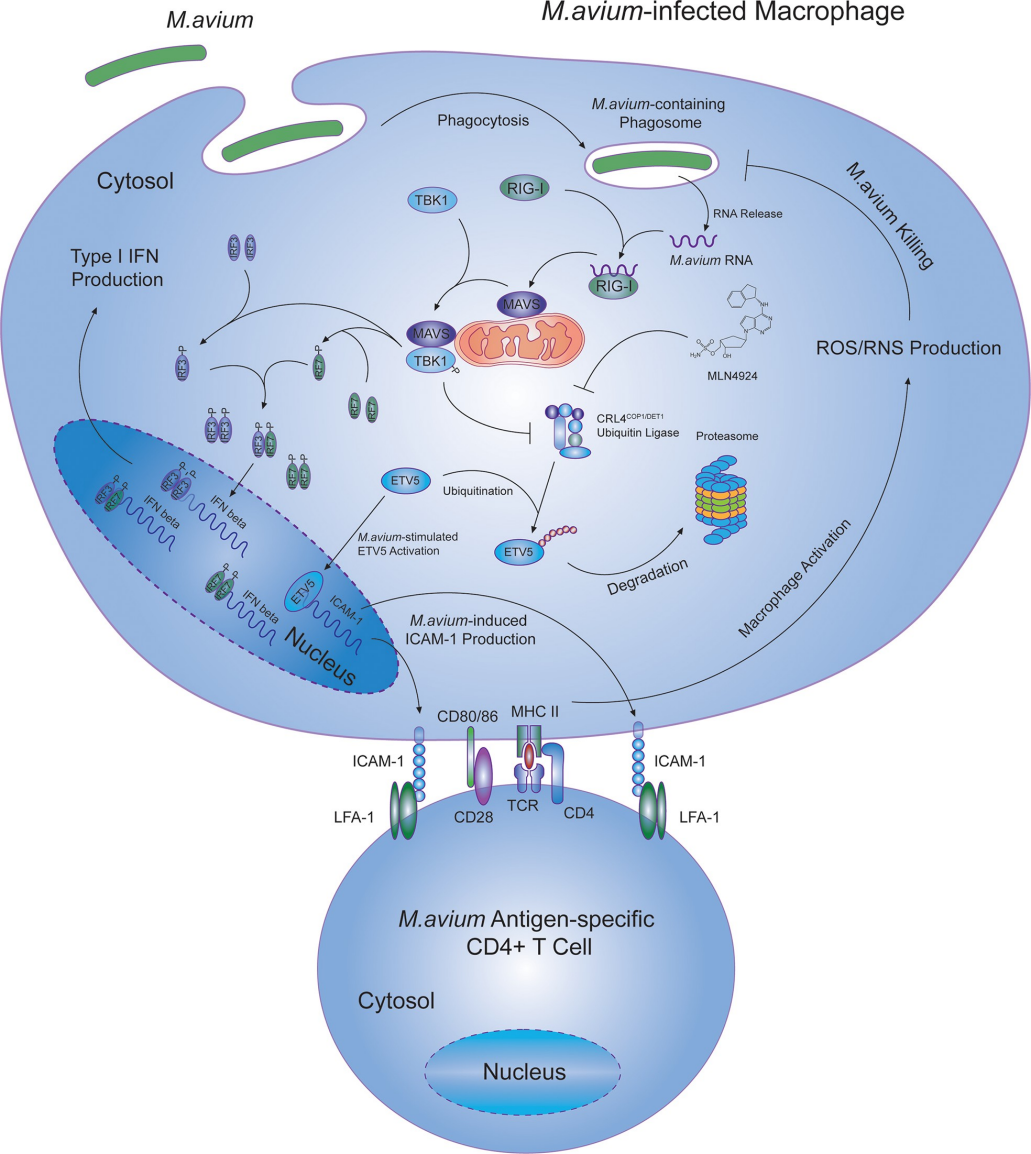
Host cytosolic RIG-I/MAVS/TBK1 RNA sensing pathway promotes antigen-specific CD4^+^ T cell-mediated mycobacterial killing in *M*.*avium*-infected host cells.

In summary (Fig 8), our study reveals a previously undefined RIG-I/MAVS dependent mechanism for inducing ICAM-1 expression in macrophages following an *M*. *avium* infection and its role in CD4^+^ T cell-mediated mycobacterial killing. Additionally, in contrast to *M*.*tb*, we identified that activation of the RNA sensing pathway is beneficial to the host immune response to an *M*. *avium* infection. At present we lack a clear understanding as to for the differential response to activation of RIG-1/MAVS following the two different mycobacterial infections. To address this question additional studies are needed to determine which host factors are differentially regulated by activation of RIG-1/MAVS during an *M*.*tb* and *M*. *avium* infection including how ICAM-1 expression is induced during an *M*.*tb* infection.

## Materials and methods

### Mice

Wild-type C57BL/6 and *Mavs*^*–/–*^mice have been described previously^5^. *ICAM-1*^*–/–*^mice on a C57BL/6 background were purchased from Jackson Laboratory (Stock No: 002867). All mice were housed and bread at the institutional animal facility under specific pathogen-free conditions. Mice were housed with standard housing in cages containing 3–5 mice per cage.

### Ethic statement

The University of Notre Dame is accredited through the Animal Welfare Assurance (#A3093-01). All animal experiments were approved by the University of Notre Dame’s Institutional Animal Care and Use Committees (IACUCs) (protocol approval number # 17-02-3630). Isoflurane was used as the mouse anesthesia and mice were killed by cervical dislocation following isoflurane inhalation.

### Bacterial strains

*M*.*avium* 104 and 2151, gentamycin-resistant *M*.*avium* 104 and tdTomato-expressing *M*.*avium* (a generous gift from Dr. Luiz E. Bermudez, Oregon State University) were grown in Middlebrook 7H9 broth (Cat. No.271310; BD) supplemented with 10% (v/v) Middlebrook oleic acid–albumin–dextrose–catalase (OADC) (Cat. No. 211886; BD) and 0.2% glycerol until mid-exponential phase, and washed with complete medium for macrophages or PBS plus 0.05% Tween-80 when required.

### Cell culture

Bone marrow-derived Macrophages (BMMs) were prepared from wild type C57BL/6, *Mavs*^–/–^and *ICAM-1*^–/–^(female, 6–8 weeks) as described previously [30], and cells were grown in DMEM supplemented with 10% (v/v) heat-inactivated FBS, 20% L929 cell-conditional medium as a source of macrophage colony-stimulating factor and 100 U/ml penicillin and 100 U/ml streptomycin (SV30010, HyClone) at 37°C and 5% CO_2_. Mouse alveolar macrophages (AMs) were prepared as described previously [31], and cultured in RPMI-1640 medium supplemented with 10% (v/v) heat-inactivated FBS and 100 U/ml penicillin and 100 U/ml streptomycin.

### siRNA transfection

Mouse BMMs (3 x 10^5^ cells/well) were transfected with AllStars Negative Control siRNA (Cat.1027280, Qiagen), RIG-I (5’- GAAGCGUCUUCUAAUAAUU-3’), TBK1 (SMARTpool: ON-TARGETplus Tbk1 siRNA, Dharmacon), IRF3 (ON-TARGETplus Mouse Irf3 siRNA, Dharmacon), IRF7 (SMARTpool: ON-TARGETplus Irf7 siRNA, Dharmacon), or ETV5 (SMARTpool: ON-TARGETplus Etv5 siRNA, Dharmacon) siRNA oligos (25 pmol/3 x 10^5^ cells) in 24-well plates using Lipofectamine 2000 (Cat.11668-027, Invitrogen) following the manufacturer’s protocol. BMM were maintained for 48 hr after transfection at 37°C, 5% CO_2_ in complete medium before use in the various experiments.

### RNA purification

To measure mycobacterial RNA in the cytosol of *M*.*avium*-infected macrophages, cytosolic RNA was purified as described previously^5^. For the measurement of host cytokine and ETV5 mRNA, BMMs were infected with *M*.*avium* at an MOI of 10 at 37°C and 5% CO_2_ for various time as indicated and then total cellular RNA was purified using Qiagen RNeasy Plus Mini Kit (Cat.No. 74136, Qiagen).

### qRT-PCR

RNAs were firstly treated with DNase I (Cat.18068015, Invitrogen) following the manufacturer’s introduction. For mycobacterial RNA, cDNA were synthesized with AMV Reverse Transcriptase (Cat.M0277, NEB) and a mixture of *M*.*avium* reverse primers. *mce1B*: Forward, 5’-TCGTGGTGTTCGGTCAGATG-3’; Reverse, 5’-GAACTGACCTTGCCGACCTC-3’, *rpoC*: Forward, 5’-CAATTCAGCTGCACCCGTTG-3’; Reverse, 5’-GTTGGACGACAGCATCAGGA-3’, *polA*: Forward, 5’-CATCAACTCCACCTCGCTGT-3’; Reverse, 5’-CGAAATCGCTCTGCAACTCG-3’, and *MAV4436*: Forward, 5’- GAACGACGGCATGAAATCGC-3’; Reverse, 5’-TCGATGGTGAGCTGATCGAA-3’. For the host cytokine and ETV5 analysis, cDNA was synthesized using AMV Reverse Transcriptase and Oligo(dT)_20_ primer. IFN-β: Forward, 5’-TCCGAGCAGAGATCTTCAGGAA-3’; Reverse, 5’- TGCAACCACCACTCATTCTGAG-3’, ICAM-1: Forward, 5’-TTTCCGGAGAGTGTGGAGCT-3’; Reverse, 5’-GAGCAGCACTGCTGAGAGCT-3’. DPPIV: Forward,5’- CAATAGTTCTGCTGAGCAAAGA-3’; Reverse, 5’- TTGTTTGTAGAGGTATTCAAAGTC-3’, CHI3L1: Forward,5’- TCCAGAGCTGCTCTGCGTACAA-3’; Reverse, 5’- GCTGATGTTGGCAAAGCTGTA-3’, TNFRSF11B: Forward,5’- CACCTACCTAAAACAGCACTG-3’; Reverse, 5’- TCACGGACTGCAGTTCCTTG-3’, Complement Factor D: Forward,5’- TACATGGCTTCCGTGCAAGTG-3’; Reverse, 5’- TGCACAGAGTCGTCATCCGT-3’, ETV5: Forward, 5’- CAGGCTGTACTTTGATGATACTT-3’; Reverse, 5’- GTCATCCAGGAGGGTGACAA-3’. Quantitative PCR was performed using PerfeCTa SYBR® Green SuperMix (Cat. 95054, Quantabio) and primers as described above on StepOnePlus Real-Time PCR System (Applied Biosystems). GAPDH: Forward, 5’-TCGTCCCGTAGACA AAATGG-3’; Reverse: 5’-TTGAGGTCAATGAAGGGGTC-3’, was used as an input control.

### Whole-cell lysates and nuclear fraction preparation

BMMs were left uninfected or infected with *M*.*avium* 104 at an MOI of 10 for 24 hr at 37°C and 5% CO2, and then washed with pre-cold PBS three time. Whole-cell lysates (WCL) and nuclear fraction were prepared as described previously [5].

### Western blot

WCL and nuclear fraction were denatured at 95°C for 10 min and separated by 12.0% SDS-PAGE gel. Proteins were transferred onto nitrocellulose membranes and probed with anti-IRF3 (Cat.A303-384A, Bethyl Laboratories Inc), anti-IRF7 (Cat. 3941; Prosci Inc.), anti-TBK1 (Cat.3504, Cell Signaling Technology), anti-phospho-TBK1 (Ser172) (Cat.5483, Cell Signaling Technology), anti-NF-κB (Cat. 8242T, Cell Signaling Technology), anti-Ets-2 (cat. sc-365666, SCBT), anti-ETV5 (Cat. sc-100941, SCBT), anti-Stat1 (Cat. sc-591, SCBT), anti-AP-2(Cat. sc-12726, SCBT), anti-SP1(Cat. sc-17824, SCBT), anti-iNOS (Cat. 610599, BD Bioscience), anti-β-actin (Cat.4970, Cell Signaling Technology), and anti-Histone H3 (Cat.9717, Cell Signaling Technology) antibodies, followed by goat anti-rabbit IgG-HRP (Cat.31460, Thermo Scientific) or goat anti-mouse IgG-HRP (Cat.31438, Thermo Scientific).

### E3 Ubiquitin ligase CRL4^COP1/DET1^ inhibition assay

For qRT-PCR and western blot analysis, MLN4924 (Cat. 905579-51-3, Cayman Chemical) was dissolved in DMSO and added at the time *M*.*avium* infection into cell culture media to the specified concentrations. For confocal microscopy analysis and *M*.*avium* survival assay in BMM-T cell coculture system, MLN4924 was added at the time of *M*.*avium* infection into BMM culture at a final centration of 0.5 μM and removed by three washes with complete BMM medium prior to adding CD4^+^ T cells.

### Mouse infection assay

*Mavs*^*–/–*^and wild-type C57BL/6 mice (8–10 weeks old, female) were intratracheally infected with *M*.*avium* 104 at a dose of 1X10^7^ CFU as described previously [32]. *M*.*avium* input in the lung of mice was determined at 5 hr post-infection. At 1, 2, 3, 5, 8 and 12 weeks post-infection, mouse serum was obtained/prepared via cardiac puncture and BD Microtainer Serum Separator Tube. Mouse lungs and spleens were also harvested, homogenized, and plated onto Middlebrook 7H10 agar plates, and mycobacterial colonies were counted after 10–14 days of incubation at 37 ^o^C. For pathological analysis, mouse lung sections were prepared and stained with H&E at the Histology Core Facility of University of Notre Dame, and histopathological score was evaluated as described previously [33]. *M*.*avium* infection was carried out in the biosafety level 2 laboratory. Isoflurane was used as the mouse anesthesia and mice were killed by cervical dislocation following isoflurane inhalation.

### Mouse cytokine array analysis

Mouse blood was harvested by cardiac puncture at 3 weeks post *M*.*avium* infection and serum was prepared using BD Microtainer Serum Separator Tube as described above. The abundance of mouse cytokines in serum was analyzed using Proteome Profiler Mouse XL Cytokine Array (Cat. ARY028, R&D Systems) according to manufacturer’s instruction. The intensity of the spots was determined by ImageJ software.

### Survival assay of *M*.*avium* in mouse BMMs and AMs

Cells were infected with *M*.*avium* 104 at an MOI of 10 for 1 hr at 37°C and 5% CO_2_, and then washed with complete medium thrice, and incubated for another 24 and 72 hr at 37°C and 5% CO_2_. At the given time, cells were washed with pre-cold PBS thrice and lysed in 0.05% SDS. A series of dilution of cell lysates in PBS (1x) were added onto 7H10 agar plates supplemented with 10% (v/v) OADC and 0.2% glycerol. Plates were incubated at 37°C for 10–15 days until counting.

### *M*.*avium* survival assay in BMMs cocultured with T cell

Mouse BMMs were differentiated as described above in complete DMEM medium. Seven days after incubation, cells were washed and resuspended in BMM media and seeded into 12-well plates (1x10^5^ cells/well) overnight at 37°C and 5% CO_2_. BMMs were then infected with *M*.*avium* strains for 1 hr at an MOI of 10 at 37°C and 5% CO_2_ and subsequently washed with complete RPMI-1640 medium thrice. Infected cells were further incubated at 37°C and 5% CO_2_ overnight. These cells were used for the BMM-T cell coculture assay. Mouse CD4^+^ T cells and CD8^+^ T cells were isolated from the lung of uninfected and *M*.*avium*-infected WT or *Mavs*^*-/-*^ mice (3 weeks post-intratracheal infection as described above). T cells were isolated using MojoSort™ Mouse CD4^+^ T Cell Isolation Kit (Cat. 480005, Biolegend) and MojoSort™ and mouse CD8+ T Cell Isolation Kit (Cat. 480007, Biolegend). Purified CD4^+^ T cells and CD8^+^ T cells were added to the *M*.*avium*-infected BMMs culture and incubated for 1, 24 and 72 hr at 37°C and 5% CO_2_. Cocultured cells were washed with pre-cold PBS thrice and then lysed in 0.05% SDS and spread on 7H10 agar plates containing 10% OADC. Plates were incubated at 37°C for 10–15 days until counting and results were expressed as Log_10_.

### Triple-cell population coculture assay

A two-cell population (BMM-T cells) coculture was first established as described above, Briefly, WT BMMs were initially infected with Gen^S^ *M*.*avium* at MOI of 10 for 24 hr at 37°C and 5% CO_2_ and then cocultured with CD4^+^ T cells isolated from WT *M*.*avium*-infected C57B/6 mice (isolated from mice 3 weeks post-infection). 24 hr after the addition of CD4+ T cells to the infected macrophages this two-cell population was used for Triple-cell Population Coculture Assay. WT, *Mavs*^*-/-*^ and *ICAM-1*^*-/-*^ BMMs were infected with Gen^R^ *M*.*avium* at an MOI of 10 for 1 hr at 37°C and 5% CO_2_. Cells were then washed with complete medium thrice, and incubated for another 24 at 37°C and 5% CO_2_. Subsequently, these infected WT, *Mavs*^*-/-*^ and *ICAM-1*^*-/-*^ BMMs were added into the two-cell population coculture at a ratio: Gen^S^ *M*.*avium*-infected BMMs: Gen^R^ *M*.*avium*-infected BMMs:T cells = 4:1:10. Mixed cells were incubated for another 1, 24 and 72 hr at 37°C and 5% CO_2_. Cocultured cells were washed with pre-cold PBS thrice and then lysed in 0.05% SDS. Gen^R^ *M*.*avium* CFU was determined using 7H10 agar plates supplemented with gentamycin (200μg/ml) and 10% OADC. Total *M*.*avium* CFU was determined using 7H10 agar plates plus 10% OADC. Gen^S^ *M*.*avium* CFU was calculated by the formula: total *M*.*avium* CFU—Gen^R^ *M*.*avium* CFU.

### Flow cytometry analysis

For BMMs, cells were uninfected or infected with *M*.*avium* 104 at a MOI of 10 for 24 hr at 37°C and 5% CO_2_, and then stained with FITC anti-mouse ICAM-1 (Cat. 116105, Biolegend), FITC anti-mouse I-Ab (Cat. 116405, Biolegend), FITC anti-mouse CD80 (Cat. 104705, Biolegend), FITC anti-mouse CD86 (Cat. 105005, Biolegend) or FITC anti-mouse CD40 (Cat. 124607, Biolegend) as described previously [34]. For mouse lung cells, WT or *Mavs*^*-/-*^ mice were infected with WT *M*.*avium* as described above and lungs were harvested 3 weeks post infection. Single lung cells were prepared [34] and stained using PE-conjugated anti-mouse CD4 (Cat.100408, Biolegend), or CD8 (Cat. 553033, BD Biosciences) Abs, APC-conjugated anti-mouse F4/80 (Cat. No. 123115, Biolegend), CD11c (Cat. No. 117309, Biolegend) or Ly-6G (Cat. No. 127613, Biolegend) Abs, FITC-conjugated anti-mouse INF-γ (Cat. 505806, Biolegend), or FITC-conjugated anti-mouse ICAM-1 Abs. For in vitro T cell proliferation assay, CD4^+^ T cells isolated from WT C57BL/6 mice following a 3 week infection with WT M. avium 104, were labeled with Vybrant® CFDA SE Cell Tracer Kit (Cat. V12883, Invitrogen) and then cocultured with WT or *Mavs*^*-/-*^ BMMs that were uninfected or infected with *M*.*avium* 104 (two-cell population coculture). The BMMs and T cells were incubated together for 72 hr at 37°C and 5% CO_2_. All samples were analyzed by BD LSRFortessa X-20 cytometer.

### Confocal microscopy analysis for macrophage-T cell synapse

C57BL/6, *Mavs*^*-/-*^ or *ICAM-1*^*-/-*^ BMMs were infected with an *M*.*avium* strain expressing tdTomato at an MOI of 10. Twenty-four hours post infection, infected cells were cocultured with CD4^+^ T cells isolated from WT *M*.*avium*-infected mice at a ratio (BMM:T cell) of 1:2. Twenty-four hours post incubation, cells were gently washed with precold PBS thrice, and fixed in 2% PFA for 15 min at RT, and then permeabilized in 0.1% Triton X-100 in PBS for 15 minutes at RT. Cells were blocked in PBS containing 2% FBS and further stained with Alexa Fluor™ 647 phalloidin (Cat. A22287, Invitrogen) and Alexa Fluor 488 anti-mouse CD4 Antibody (Cat. No. 100529, Biolegend), and analyzed by Nikon C2+ confocal laser scanning microscope. For quantitative analysis, at least 100 BMMs per condition were counted in multiple random areas of the slide. Data was normalized to the number of T cells conjugated to M.avium-infected WT BMMs for one biological sample, which was set to 100%.

### Alveolar macrophage adoptive transfer assay

Mouse AMs were isolated from WT, *Mavs*^*-/-*^ or *ICAM-1*^*-/-*^ mice (Female, 8 to 10 weeks old) as described above, and then infected with Gen^R^ *M*.*avium* strains at an MOI of 10 for 24 hr at 37°C and 5% CO_2_. Infected AMs were washed with pre-cold PBS thrice and then intratracheally injected into mice at a dose of 2x10^5^ cells/mouse in 15 μl PBS. At the time of AM injection, recipient mice had been infected with Gen^S^ *M*.*avium* for 3 weeks as described [32]. At day 1, 3 and 6 post injection, mouse lungs were harvested, homogenized, and spread on 7H10 plus 10% OADC with or without gentamycin (200μg/ml). Bacterial number was counted 10–15 days after incubation at 37°C. For AM survival assay in mice after adoptive transfer, Gen^R^ *M*.*avium*-infected AMs were labeled with Vybrant® CFDA SE Cell Tracer Kit before intratracheal injection. Single mouse lung cells were prepared as described previously at day 1, 3 and 6 post injection^34^, and analyzed by BD LSRFortessa X-20 cytometer.

### Intracellular ROS measurement

Mouse WT, *Mavs*^*-/-*^ or *ICAM-1*^*-/-*^ BMMs were infected with WT *M*.*avium* at an MOI of 10 for 24 hr at 37°C and 5% CO_2_, and then cocultured with/without CD4^+^ T cells isolated from WT mice (Female, 8 to 10 weeks old) previously infected with WT *M*.*avium* for 3 weeks. Intracellular ROS concentration in infected BMMs was measured at various time points post coculture using Fluorometric Intracellular Ros Kit (Cat. MAK142-1KT, Sigma-Aldrich) following the manufacturer’s instruction. The fluorescence intensity was measured using BioTek Synergy XL microplate reader.

### ELISA

IFN-β production in mouse serum or BMMs culture supernatants at 24 or 72 hr post *M*.*avium* infection at 37°C and 5% CO_2_ was measured. ELISA was performed according to the manufacturer’s instruction (ebioscience). Capture antibody (Purified anti-mouse IFN-β Antibody, Cat. 519202, Biolegend), detection antibody (Biotin anti-mouse IFN-β Antibody, Cat. 508105, Biolegend), IFN-β standard (Cat. 581309, Biolegend), Avidin-HRP (Cat.18-4100-94, ebioscience), and TMB (Cat.00-4201-56, ebioscience). IFN-γ in culture supernatant and lung homogenate was measured using IFN gamma ELISA kit (Cat. 88-7314-22; eBioscience). For in vivo IFN-γ measurement, mouse lung homogenate was prepared as described previously [35].

### Statistical analysis

Statistical analysis was performed to determine differences between groups by two-tailed Student’s t-tests or Mann–Whitney U test using GraphPad Prism software (Version 5.04, Graphpad Software). P-value < 0.05 was considered significant.

## Supporting information

S1 Fig Western blot analysis for siRNA efficiency in mouse BMMs. A) RIG-I; B) TBK1; C) IRF3 and D) IRF7. β)actin served as a load control. Mock, untreated; Control, negative control siRNA. Data shown are representative of three independent experiments.
(PPTX)

S2 Fig Flow cytometry and ELISA analysis for cytokine production in immune cells and mice post *M*.*avium* infection. (A) Flow cytometry analysis for ICAM-1 production on lung immune cells isolated from WT and *Mavs*^*-/-*^ mice infected with WT *M*.*avium* for 3 weeks. (B) Similar to A), but IFN-γ production on CD4^+^ and CD8^+^ T cells. (C) ELISA analysis for IFN-γ production in mouse lung after 3 weeks post *M*.*avium* infection. The data shown is the combination of three independent experiments (biological repeats). n = 3 per group each experiment. n.s., not statistically significant by Mann–Whitney U test. (D) MHCII, CD80, CD86 and CD40 abundance on WT and *Mavs*^*-/-*^ BMMs infected with *M*.*avium* at 24 hr post infection. Data shown in A, B and D are representative of at least three independent experiments.
(PPTX)

S3 Fig *M*.*avium* burden in BMMs or BMMs cocultured with CD4^+^/CD8^+^ T cells. (A) *M*.*avium* burden in WT and *Mavs*^*-/-*^ BMMs at 1, 24 and 72 hr post infection. (B) *M*.*avium* burden in mouse BMMs pretreated with negative control siRNA or RIG-I-, TBK1-, IRF3-, or IRF7-specific siRNA. (C) *M*.*avium* burden in WT and *Mavs*^*-/-*^ BMMs cocultured with/without CD8^+^ T cells isolated from WT *M*.*avium*-infected mice (BMM:T cells = 1:2). (D) *M*.*avium* burden in WT BMMs cocultured with CD4^+^ or CD8^+^ T cells isolated from nared with CD4negative control siR Data shown are the mean ared with CD4negative control siRNA or RIG-I-, TBK1-, IRF3-, or IRF7-specific siRNA.group each experiment. n.s., not statistically significant by Mann–Whitney U test.as expre***P < 0.001 by Student’s t-test (two-tailed).
(PPTX)

S4 Fig Survival of *M*. *avium-*infected and CFSE-labeled mouse AMs in the *M*. *avium*-infected mouse lung post adoptive transfer. (A) Cell number of *M*. *avium*-infected WT and *Mavs*^*-/-*^ AMs in the lung of *M*. *avium*-infected WT mice over time post AM injection. (B) Cell number of *M*. *avium*-infected WT and *ICAM-1*^*-/-*^ AMs in the lung of *M*. *avium*-infected WT mice over time post AM injection. The data shown is the combination of two independent experiments. n = 3 mice per group each experiment.
(PPTX)
