# Supplementary material for: Host cytosolic RNA sensing pathway promotes T Lymphocyte-mediated mycobacterial killing in macrophages
Source: PLoS Pathog. 2020 May 28;16(5):e1008569. doi: 10.1371/journal.ppat.1008569 (PMC7282665; doi:10.1371/journal.ppat.1008569)
Supplement: S1 Fig — A) RIG-I; B) TBK1; C) IRF3 and D) IRF7. β)actin served as a load control. Mock, untreated; Control, negative control siRNA. Data shown are representative of three independent experiments. (PPTX) [file ppat.1008569.s001.pptx]

## Slide 1
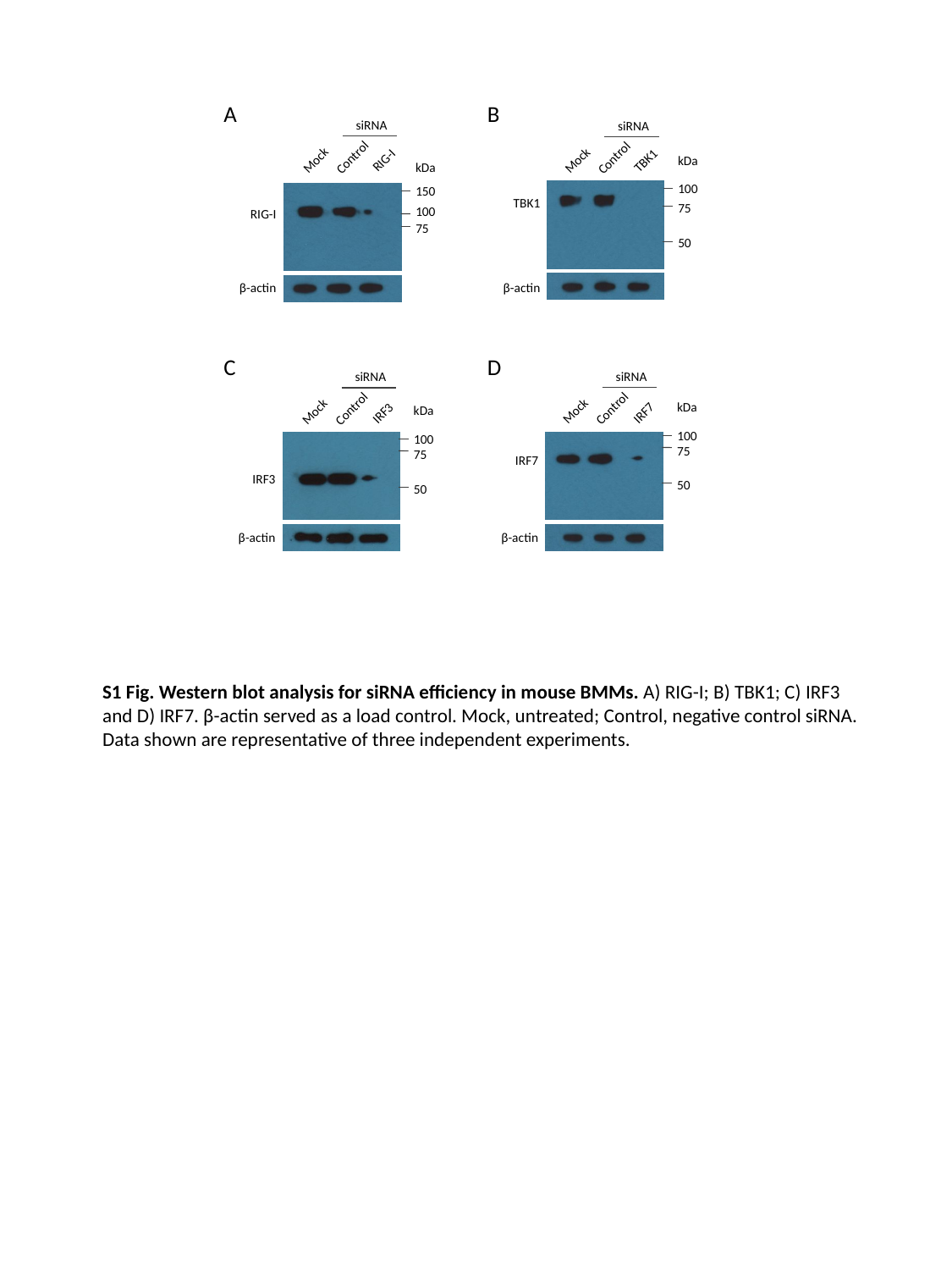

A
B
siRNA
Control
Mock
RIG-I
siRNA
Control
Mock
TBK1
kDa
100
75
50
kDa
150
TBK1
β-actin
100
RIG-I
β-actin
75
C
D
siRNA
Control
Mock
IRF7
siRNA
Control
Mock
IRF3
kDa
100
75
50
kDa
100
75
50
IRF7
β-actin
IRF3
β-actin
S1 Fig. Western blot analysis for siRNA efficiency in mouse BMMs. A) RIG-I; B) TBK1; C) IRF3 and D) IRF7. β-actin served as a load control. Mock, untreated; Control, negative control siRNA. Data shown are representative of three independent experiments.
