# Supplementary material for: Host cytosolic RNA sensing pathway promotes T Lymphocyte-mediated mycobacterial killing in macrophages
Source: PLoS Pathog. 2020 May 28;16(5):e1008569. doi: 10.1371/journal.ppat.1008569 (PMC7282665; doi:10.1371/journal.ppat.1008569)
Supplement: S2 Fig — (A) Flow cytometry analysis for ICAM-1 production on lung immune cells isolated from WT and Mavs-/- mice infected with WT M.avium for 3 weeks. (B) Similar to A), but IFN-γ production on CD4+ and CD8+ T cells. (C) ELISA analysis for IFN-γ production in mouse lung after 3 weeks post M.avium infection. The data shown is the combination of three independent experiments (biological repeats). n = 3 per group each experiment. n.s., not statistically significant by Mann–Whitney U test. (D) MHCII, CD80, CD86 and CD40 abundance on WT and Mavs-/- BMMs infected with M.avium at 24 hr post infection. Data shown in A, B and D are representative of at least three independent experiments. (PPTX) [file ppat.1008569.s002.pptx]

## Slide 1
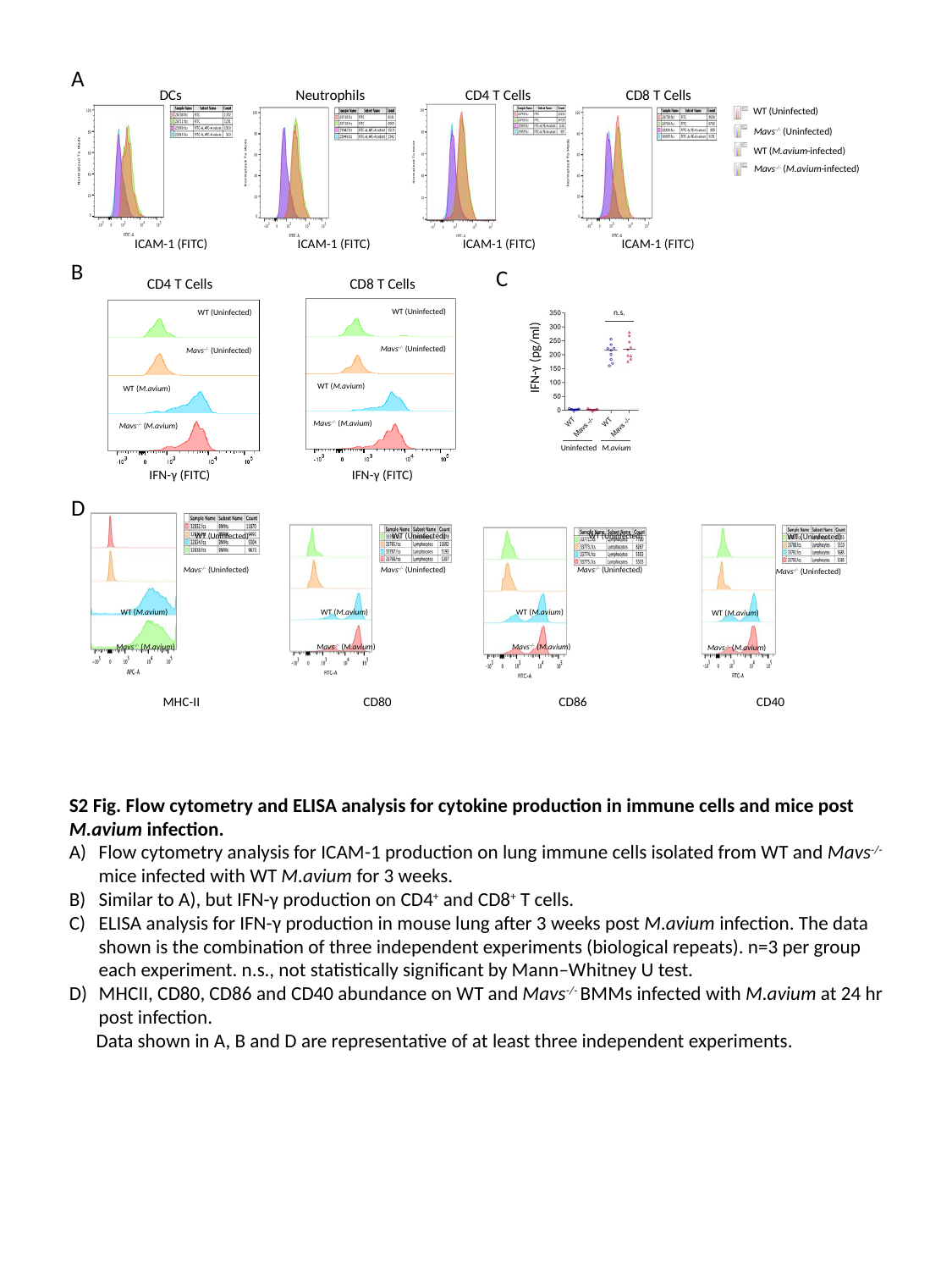

A
DCs
Neutrophils
CD4 T Cells
CD8 T Cells
WT (Uninfected)
Mavs-/- (Uninfected)
WT (M.avium-infected)
Mavs-/- (M.avium-infected)
ICAM-1 (FITC)
ICAM-1 (FITC)
ICAM-1 (FITC)
ICAM-1 (FITC)
B
C
CD4 T Cells
CD8 T Cells
WT (Uninfected)
WT (Uninfected)
n.s.
Mavs-/- (Uninfected)
Mavs-/- (Uninfected)
IFN-γ (pg/ml)
WT (M.avium)
WT (M.avium)
Mavs-/- (M.avium)
Mavs-/- (M.avium)
M.avium
Uninfected
IFN-γ (FITC)
IFN-γ (FITC)
D
WT (Uninfected)
Mavs-/- (Uninfected)
WT (M.avium)
Mavs-/- (M.avium)
MHC-II
WT (Uninfected)
Mavs-/- (Uninfected)
WT (M.avium)
Mavs-/- (M.avium)
CD80
WT (Uninfected)
Mavs-/- (Uninfected)
WT (M.avium)
Mavs-/- (M.avium)
CD40
WT (Uninfected)
Mavs-/- (Uninfected)
WT (M.avium)
Mavs-/- (M.avium)
CD86
S2 Fig. Flow cytometry and ELISA analysis for cytokine production in immune cells and mice post M.avium infection.
Flow cytometry analysis for ICAM-1 production on lung immune cells isolated from WT and Mavs-/- mice infected with WT M.avium for 3 weeks.
Similar to A), but IFN-γ production on CD4+ and CD8+ T cells.
ELISA analysis for IFN-γ production in mouse lung after 3 weeks post M.avium infection. The data shown is the combination of three independent experiments (biological repeats). n=3 per group each experiment. n.s., not statistically significant by Mann–Whitney U test.
MHCII, CD80, CD86 and CD40 abundance on WT and Mavs-/- BMMs infected with M.avium at 24 hr post infection.
 Data shown in A, B and D are representative of at least three independent experiments.
