# Supplementary material for: Host cytosolic RNA sensing pathway promotes T Lymphocyte-mediated mycobacterial killing in macrophages
Source: PLoS Pathog. 2020 May 28;16(5):e1008569. doi: 10.1371/journal.ppat.1008569 (PMC7282665; doi:10.1371/journal.ppat.1008569)
Supplement: S3 Fig — (A) M.avium burden in WT and Mavs-/- BMMs at 1, 24 and 72 hr post infection. (B) M.avium burden in mouse BMMs pretreated with negative control siRNA or RIG-I-, TBK1-, IRF3-, or IRF7-specific siRNA. (C) M.avium burden in WT and Mavs-/- BMMs cocultured with/without CD8+ T cells isolated from WT M.avium-infected mice (BMM:T cells = 1:2). (D) M.avium burden in WT BMMs cocultured with CD4+ or CD8+ T cells isolated from nared with CD4negative control siR Data shown are the mean ared with CD4negative control siRNA or RIG-I-, TBK1-, IRF3-, or IRF7-specific siRNA.group each experiment. n.s., not statistically significant by Mann–Whitney U test.as expre***P < 0.001 by Student’s t-test (two-tailed). (PPTX) [file ppat.1008569.s003.pptx]

## Slide 1
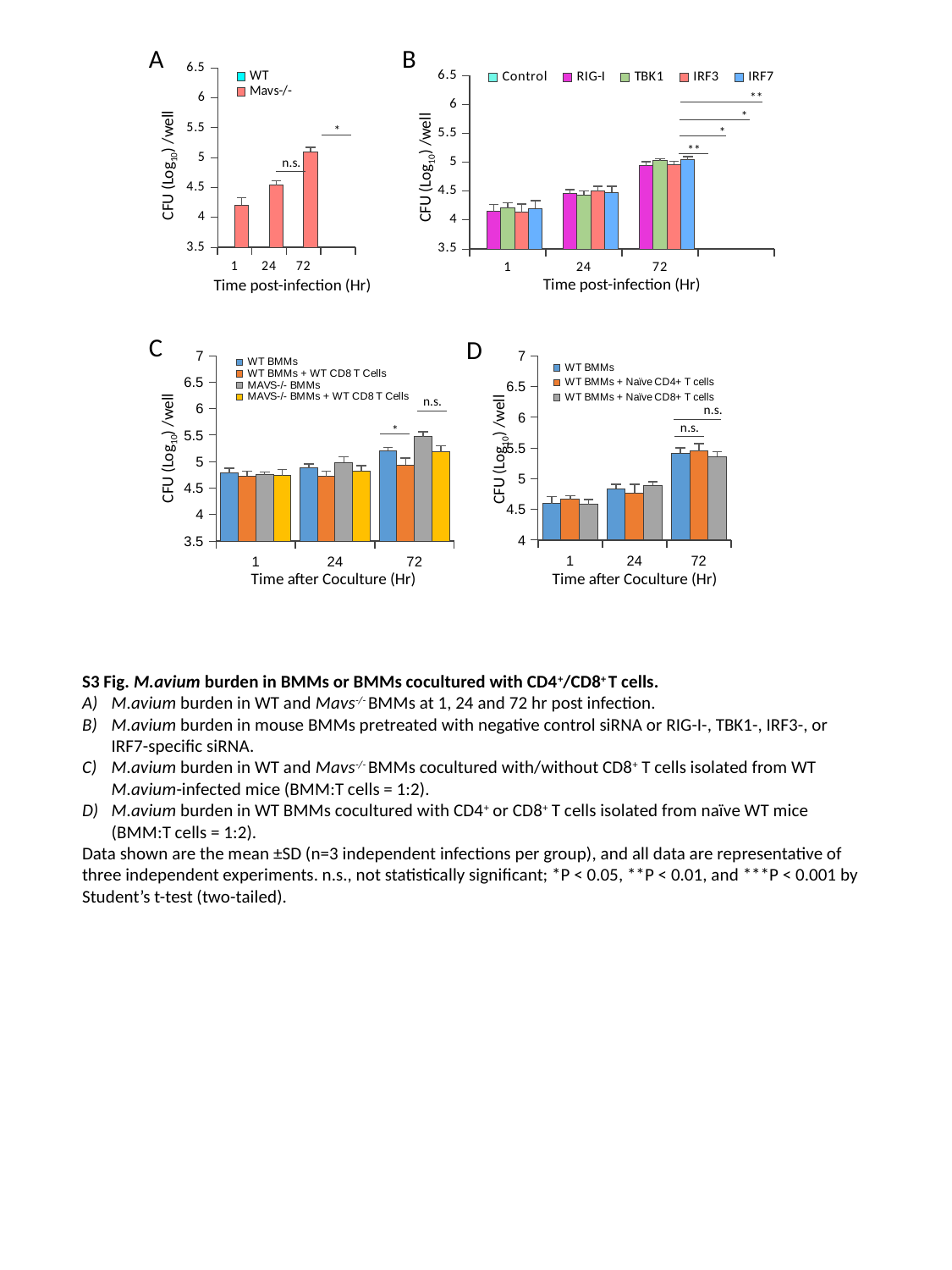

A
B
### Chart
| Category | WT | Mavs-/- |
|---|---|---|
| 1 | 4.16449690880579 | 4.21022603357284 |
| 24 | 4.453712453588096 | 4.536820177784798 |
| 72 | 4.854275679264988 | 5.0999272779149765 |
### Chart
| Category | Control | RIG-I | TBK1 | IRF3 | IRF7 |
|---|---|---|---|---|---|
| 1 | 4.20431644458603 | 4.152928447494436 | 4.210861601586468 | 4.137101323410007 | 4.202800568669122 |
| 24 | 4.396485073537888 | 4.455063301591829 | 4.432041690303625 | 4.503891505115861 | 4.480521067526934 |
| 72 | 4.748658565003741 | 4.941674245097032 | 5.02705433096195 | 4.961889292573873 | 5.040602738612268 |**
*
*
*
**
n.s.
CFU (Log10) /well
CFU (Log10) /well
Time post-infection (Hr)
Time post-infection (Hr)
C
D
### Chart
| Category | WT BMMs | WT BMMs + WT CD8 T Cells | MAVS-/- BMMs | MAVS-/- BMMs + WT CD8 T Cells |
|---|---|---|---|---|
| 1 | 4.780361874724039 | 4.728960440943031 | 4.751190906741804 | 4.731586960767028 |
| 24 | 4.8762506736182525 | 4.721635779322425 | 4.979372714782412 | 4.825097905794191 |
| 72 | 5.2053468035784105 | 4.934760186456836 | 5.472476507621361 | 5.183687447632292 |
### Chart
| Category | WT BMMs | WT BMMs + Naïve CD4+ T cells | WT BMMs + Naïve CD8+ T cells |
|---|---|---|---|
| 1 | 4.59631239235127 | 4.66226015961064 | 4.581094367417698 |
| 24 | 4.834528629755413 | 4.765931565134167 | 4.887427582243851 |
| 72 | 5.416933092414484 | 5.452698147453655 | 5.363218207101302 |n.s.
n.s.
n.s.
*
CFU (Log10) /well
CFU (Log10) /well
Time after Coculture (Hr)
Time after Coculture (Hr)
S3 Fig. M.avium burden in BMMs or BMMs cocultured with CD4+/CD8+ T cells.
M.avium burden in WT and Mavs-/- BMMs at 1, 24 and 72 hr post infection.
M.avium burden in mouse BMMs pretreated with negative control siRNA or RIG-I-, TBK1-, IRF3-, or IRF7-specific siRNA.
M.avium burden in WT and Mavs-/- BMMs cocultured with/without CD8+ T cells isolated from WT M.avium-infected mice (BMM:T cells = 1:2).
M.avium burden in WT BMMs cocultured with CD4+ or CD8+ T cells isolated from naïve WT mice (BMM:T cells = 1:2).
Data shown are the mean ±SD (n=3 independent infections per group), and all data are representative of three independent experiments. n.s., not statistically significant; *P < 0.05, **P < 0.01, and ***P < 0.001 by Student’s t-test (two-tailed).
