# Supplementary material for: Host cytosolic RNA sensing pathway promotes T Lymphocyte-mediated mycobacterial killing in macrophages
Source: PLoS Pathog. 2020 May 28;16(5):e1008569. doi: 10.1371/journal.ppat.1008569 (PMC7282665; doi:10.1371/journal.ppat.1008569)
Supplement: S4 Fig — (A) Cell number of M. avium-infected WT and Mavs-/- AMs in the lung of M. avium-infected WT mice over time post AM injection. (B) Cell number of M. avium-infected WT and ICAM-1-/- AMs in the lung of M. avium-infected WT mice over time post AM injection. The data shown is the combination of two independent experiments. n = 3 mice per group each experiment. (PPTX) [file ppat.1008569.s004.pptx]

## Slide 1
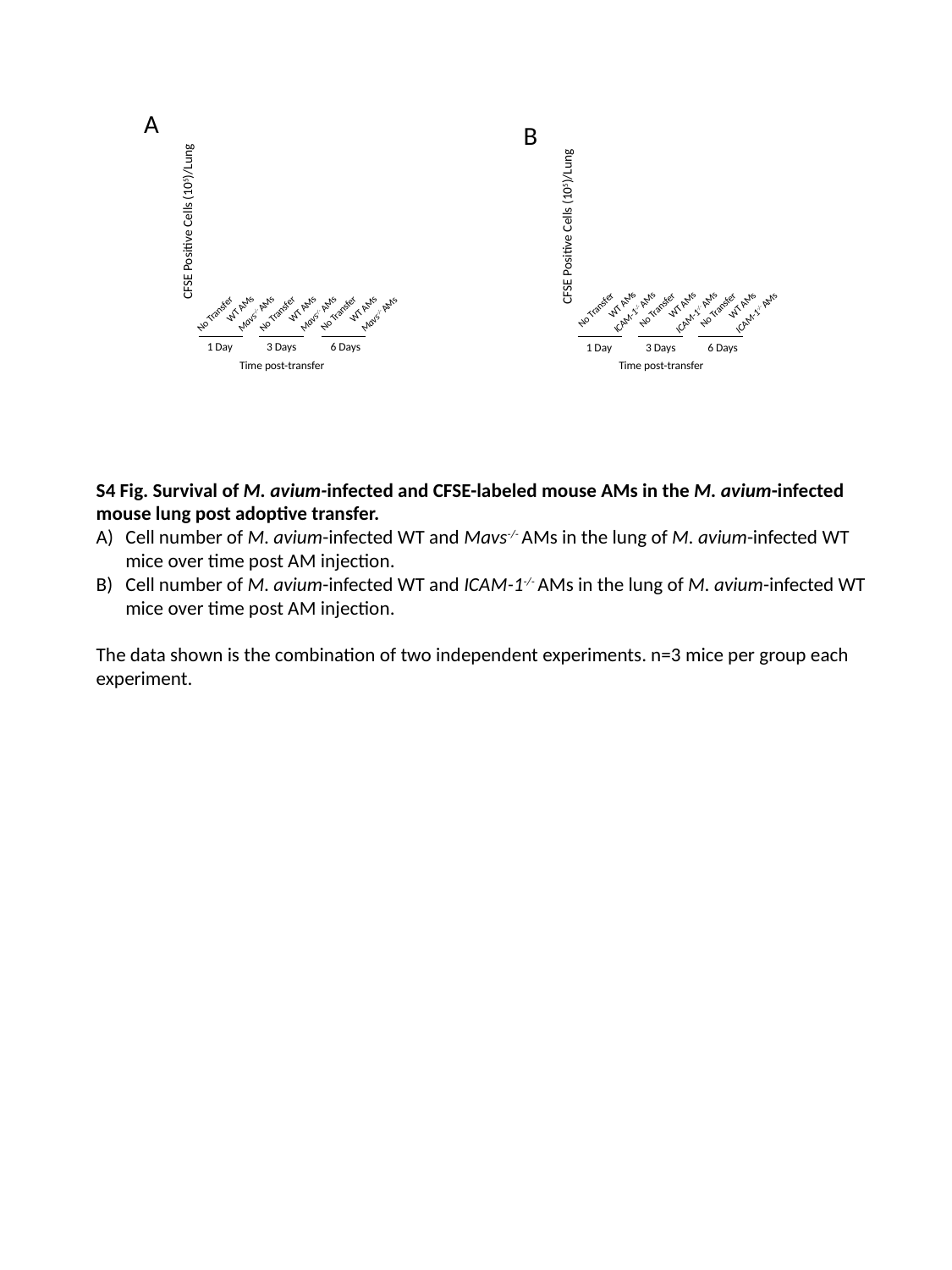

A
B
CFSE Positive Cells (105)/Lung
CFSE Positive Cells (105)/Lung
WT AMs
No Transfer
ICAM-1-/- AMs
WT AMs
No Transfer
ICAM-1-/- AMs
WT AMs
No Transfer
ICAM-1-/- AMs
WT AMs
Mavs-/- AMs
No Transfer
WT AMs
Mavs-/- AMs
No Transfer
WT AMs
No Transfer
Mavs-/- AMs
1 Day
3 Days
6 Days
Time post-transfer
1 Day
3 Days
6 Days
Time post-transfer
S4 Fig. Survival of M. avium-infected and CFSE-labeled mouse AMs in the M. avium-infected mouse lung post adoptive transfer.
Cell number of M. avium-infected WT and Mavs-/- AMs in the lung of M. avium-infected WT mice over time post AM injection.
Cell number of M. avium-infected WT and ICAM-1-/- AMs in the lung of M. avium-infected WT mice over time post AM injection.
The data shown is the combination of two independent experiments. n=3 mice per group each experiment.
